# Photochemistry with Chlorine Trifluoride: Syntheses and Characterization of Difluorooxychloronium(V) Hexafluorido(non)metallates(V), [ClOF_2_][*M*F_6_] (*M*=V, Nb, Ta, Ru, Os, Ir, P, Sb)

**DOI:** 10.1002/chem.202003629

**Published:** 2020-12-22

**Authors:** Benjamin Scheibe, Antti J. Karttunen, Florian Weigend, Florian Kraus

**Affiliations:** ^1^ Fachbereich Chemie Philipps-Universität Marburg Hans-Meerwein-Straße 4 35032 Marburg Germany; ^2^ Department of Chemistry and Materials Science Aalto University 00076 Aalto Finland

**Keywords:** chlorine trifluoride, difluorooxychloronium(V) cation, fluorine, photochemistry, single-crystal X-ray diffraction

## Abstract

A photochemical route to salts consisting of difluorooxychloronium(V) cations, [ClOF_2_]^+^, and hexafluorido(non)metallate(V) anions, [*M*F_6_]^−^ (*M*=V, Nb, Ta, Ru, Os, Ir, P, Sb) is presented. As starting materials, either metals, oxygen and ClF_3_ or oxides and ClF_3_ are used. The prepared compounds were characterized by single‐crystal X‐ray diffraction and Raman spectroscopy. The crystal structures of [ClOF_2_][*M*F_6_] (*M*=V, Ru, Os, Ir, P, Sb) are layer structures that are isotypic with the previously reported compound [ClOF_2_][AsF_6_], whereas for *M*=Nb and Ta, similar crystal structures with a different stacking variant of the layers are observed. Additionally, partial or full O/F disorder within the [ClOF_2_]^+^ cations of the Nb and Ta compounds occurs. In all compounds reported here, a trigonal pyramidal [ClOF_2_]^+^ cation with three additional Cl⋅⋅⋅F contacts to neighboring [*M*F_6_]^−^ anions is observed, resulting in a pseudo‐octahedral coordination sphere around the Cl atom. The Cl−F and Cl−O bond lengths of the [ClOF_2_]^+^ cations seem to correlate with the effective ionic radii of the *M* ^*V*^ ions. Quantum‐chemical, solid‐state calculations well reproduce the experimental Raman spectra and show, as do quantum‐chemical gas phase calculations, that the secondary Cl⋅⋅⋅F interactions are ionic in nature. However, both solid‐state and gas‐phase quantum‐chemical calculations fail to reproduce the increases in the Cl−O bond lengths with increasing effective ionic radius of *M* in [*M*F_6_]^−^ and the Cl−O Raman shifts also do not generally follow this trend.

## Graphical abstract

**Pairing up**: Compounds of the [ClOF_2_]^+^ cation, [ClOF_2_][*M*F_6_], were obtained from photochemical reactions of oxides or of metals and oxygen with chlorine trifluoride. Two different structural types were observed, depending on the effective ionic radius, *r*_eff_, of the *M* ^*V*^ ion. The Cl−O bond lengths of the [ClOF_2_]^+^ cations generally correlate with *r*_eff_(*M* ^*V*^) and O/F disorder is observed for the biggest *M* ^*V*^ ions in the [*M*F_6_]^−^ anions studied.

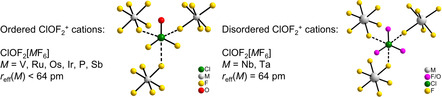

## Introduction

Chlorine oxide trifluoride, ClOF_3_,[^1^, ^2^, ^3^] is one of the currently known four stable chlorine oxyfluorides with the other three being ClO_2_F, ClO_3_F and ClO_2_F_3_.[^4^, ^5^, ^6^, ^7^, ^8^, ^9^] Chlorosyl fluoride, ClOF, is unstable towards disproportionation into ClF and ClO_2_F, and is formed as an intermediate during the hydrolysis of ClF_3_.[^10^, ^11^, ^12^, ^13^, ^14^] Perchloryl hypofluorite, ClO_4_F, does not have a Cl‐F bond and is thus different from the above mentioned oxyfluorides.[^3^, ^15^] Several synthetic routes for ClOF_3_ have been described. The first synthesis of ClOF_3_ was likely carried out by Rocketdyne in 1965, a few years before the first publications on ClOF_3_ appeared in the open literature.[^1^, ^2^, ^16^, ^17^, ^18^] It can either be synthesized by the fluorination of ClONO_2_ or of Cl_2_O at low temperatures according to Equations 1 and 2.[^2^, ^17^]

(1) $$2F_{2}+ClONO_{2}\overset{\;-35^{\circ }C\;}{\underset{}{\to }}ClOF_{3}+NO_{2}F$$

(2) $$2F_{2}+Cl_{2}O\overset{\;NaF/CsF,-78^{\circ }C\;}{\underset{}{\to }}ClOF_{3}+ClF$$

A photochemical synthesis of ClOF_3_ is also possible.[^1^, ^16^, ^18^, ^19^] ClOF_3_ can be obtained at room temperature by UV irradiation of ClF_3_/OF_2_ mixtures.^[1]^ It can also be obtained at low temperatures by irradiating mixtures of Cl_2_/F_2_/O_2_, ClF_3_/O_2_, ClO_2_F/ClF_5_, ClO_3_F/ClF_5_, ClO_3_F/F_2_, or of ClF/IOF_5_ with UV light.[^18^, ^19^] Usually a range of side products is obtained in such photochemical reactions, with yields of ClOF_3_ that are low to good with, for example, up to a 79 % yield for the mixture ClO_3_F/ClF_5_.^[19]^

ClOF_3_ shows Lewis amphoteric behavior, thus it forms adducts with both Lewis acids and Lewis bases.[^20^, ^21^, ^22^] The higher homologues, BrOF_3_ and IOF_3_, are known to exhibit similar reactivities towards Lewis acids and bases.[^23^, ^24^, ^25^, ^26^, ^27^, ^28^, ^29^, ^30^, ^31^] For example, with Lewis bases, alkali metal fluorides and ClOF_3_ form compounds containing the tetrafluoridooxidochlorate(V) anion, [ClOF_4_]^−^, which is likely tetragonal‐pyramidal like the homologous [*X*OF_4_]^−^ (*X*=Br, I) anions are.[^22^, ^23^, ^31^, ^32^] However, a crystal structure containing [ClOF_4_]^−^ anions has yet to be determined.

Compounds with the trigonal pyramidal difluoroxychloronium(V) cation, [ClOF_2_]^+^, are obtained in reactions with Lewis acids, such as the pentafluorides *M*F_5_ (Equation 3).[^20^, ^21^, ^22^, ^33^, ^34^, ^35^]

(3) $$ClOF_{3}+MF_{5}\to ClOF_{2}\left[MF_{6}\right]$$

in which *M*=P, As, Sb, V, Nb, Ta, Pt, Au, Bi, U.

The initially undesirable formation of a [ClOF_2_]^+^ salt, [ClOF_2_]_2_[SiF_6_], was also reported.[^36^, ^37^] It was obtained serendipitously by the photochemical reaction of ClF_5_/OF_2_ mixtures in quartz vessels (Equation 4).

(4) $$2CIF_{5}+2OF_{2}\overset{\;h\nu \;}{\underset{}{\to }}(ClOF_{2}{)}_{2}[SiF_{6}]+4F_{2}$$

Thus far, the only reported crystal structure of a [ClOF_2_]^+^ compound is that of [ClOF_2_][AsF_6_].^[38]^ We therefore report on the photochemical syntheses of the difluorooxychloronium(V) hexafluorido(non)metallates(V), [ClOF_2_][*M*F_6_] (*M*=V, Nb, Ta, Ru, Os, Ir, P, Sb) under UV irradiation from the reactions of the respective elements with O_2_ and ClF_3_, or with metal and nonmetal oxides and chlorine trifluoride. The products were characterized by single‐crystal X‐ray diffraction, Raman spectroscopy and quantum‐chemical solid‐state and gas‐phase calculations.

## Results and Discussion

### Photochemical syntheses of [ClOF_2_][*M*F_6_] (*M*=V, Nb, Ta, Ru, Os, Ir, P, Sb)

Our difluorooxychloronium(V) compounds have been prepared from either the photochemical reaction of ClF_3_ with metals (Os, Ir) and O_2_, or from the oxides *M*_2_O_5_ (*M*=P, V, Nb, Ta), RuO_2_⋅*x* H_2_O (*x*≈2), and Sb_2_O_4_ (Equations (5) to (8)). The starting materials were first reacted with ClF_3_ and, in the case of the metals Os and Ir, oxygen gas was also added to the reaction vessels before UV irradiation. ***Caution**! The reaction between metal powders or oxides with liquid/gaseous ClF_3_ can be vigorous to explosive*. The reaction mixtures were then irradiated with UV light from low‐pressure Hg lamps (main emission line of 254 nm) for half a day to two weeks. Crystalline difluorooxychloronium(V) compounds were obtained in all cases from ClF_3_ solutions or suspensions.

The overall reaction between Os or Ir metal with ClF_3_ and O_2_ under UV irradiation can be rationalized according to Equation 5.

(5) $$2M+7ClF_{3}+O_{2}\overset{\;h\nu \;}{\underset{}{\to }}2ClOF_{2}\left[MF_{6}\right]+5ClF$$

The initial step is likely the oxidation of the metal, followed by a Lewis acid–base reaction between excess ClF_3_ and the metal pentafluoride intermediate, to give ClF_2_[*M*F_6_] (*M*=Os, Ir). Such compounds and oxidation reactions have been previously reported for Ru, Os and Ir.[^39^, ^40^, ^41^] In a photochemical reaction, ClOF_3_ is formed, which then likely displaces [ClF_2_]^+^ as ClF_3_, resulting in the formation of the [ClOF_2_]^+^ compound. Single‐crystal X‐ray diffraction and Raman spectroscopy do not indicate the presence of [ClF_2_]^+^ compounds in the isolated products.

In the case of the oxides, the initial reaction is likely the formation of ClO_2_F and the dissolved pentafluoride, which subsequently will form a solvated [ClO_2_][*M*F_6_] (*M*=V, Nb, Ta, Ru, P, Sb) salt. The formation of ClO_2_F from the reaction of ClF_3_ with oxides such as H_2_O, *A*[ClO_3_] (*A*=Na, K), [UO_2_]F_2_ and Cs[IOF_4_] has been reported.[^10^, ^42^, ^43^, ^44^, ^45^] The Lewis base character of ClO_2_F has been previously described, where a range of Lewis acids, compounds, such as [ClO_2_][BF_4_], [ClO_2_]GeF_5_ and [ClO_2_][*M*F_6_] (*M*=Ru, P, As, Sb), were obtained.[^39^, ^46^, ^47^, ^48^, ^49^] As mentioned above, ClOF_3_ is formed in photochemical reactions, which will then likely displaces [ClO_2_]^+^ as ClO_2_F to form a [ClOF_2_]^+^ salt. Single‐crystal X‐ray diffraction and Raman spectroscopy do not indicate the presence of [ClO_2_]^+^ salts in the isolated products. The overall proposed reactions can be described in terms of Equation 6 for *M*_2_O_5_ (*M*=P, V, Nb, Ta), Equation 7 for RuO_2_⋅*x* H_2_O (*x*≈2) and Equation 8 for Sb_2_O_4_.

(6) $$2M_{2}O_{2}+14CIF_{3}\overset{\;h\nu \;}{\underset{}{\to }}4ClOF_{2}\left[MF_{6}\right]+3ClF+7ClF$$

(7) $$2RuO_{5}xH_{2}O+\left(7+x\right)ClF_{3}\overset{\;h\nu \;}{\underset{}{\to }}2ClOF_{2}\left[RuF_{6}\right]+\left(1+0.5x\right)ClO_{2}F+\left(4+0.5x\right)ClF+2xHF$$

(8) $$Sb_{2}O_{4}+7ClF_{3}\overset{\;h\nu \;}{\underset{}{\to }}2ClOF_{2}\left[SbF_{6}\right]+ClO_{2}F+4ClF$$

The thermal stabilities of [ClOF_2_]^+^ salts may be correlated with the Lewis acidity of the free parent fluoride of the anion.^[3]^ The [ClOF_2_][*M*F_6_] salts derived from AsF_5_ and SbF_5_ are more stable than those derived from VF_5_ and PF_5_.[^33^, ^34^] The aforementioned salts are more stable than [ClOF_2_]_2_[SiF_6_].[^36^, ^37^]

### Crystal structures of [ClOF_2_][*M*F_6_] (*M*=P, Sb, V, Ru, Os, Ir)

Powder X‐ray diffraction patterns were previously reported for [ClOF_2_][*M*F_6_] (*M*=V, Nb, Ta, P, As, Sb, Bi), but no lattice parameters were determined.^[34]^ Isotypism was assumed for these compounds and preliminary results of a study on single crystals of [ClOF_2_][VF_6_] indicated an orthorhombic unit cell.^[34]^ Furthermore, powder X‐ray diffraction patterns of [ClOF_2_][AsF_6_] and [ClOF_2_][PtF_6_] were indexed with orthorhombic unit cells in separate studies and the resulting lattice parameters, likely determined at room temperature, were *a=*9.94, *b=*10.78, *c=*8.16 Å, *V*≈874 Å^3^ and *a=*9.94, *b=*11.12, *c=*8.21 Å^3^, *V*≈907 Å^3^, respectively.[^21^, ^22^] In the case of [ClOF_2_][AsF_6_], the reported cell volume is a factor of ca. $\sqrt{2}$ larger than the cell volume determined by single‐crystal X‐ray diffraction at 100 K.[^22^, ^38^]

To sort out these discrepancies we determined the crystal structures of the difluorooxychloronium(V) hexafluorido(non)metallates(V), [ClOF_2_][*M*F_6_] (*M*=V, Ru, Os, Ir, P, Sb), by single‐crystal X‐ray diffraction. The compounds are isotypic with the previously described hexafluoridoarsenate(V) salt, [ClOF_2_][AsF_6_].^[38]^ They crystallize with four formula units per unit cell in the orthorhombic space group *Pna*2_1_ (No. 33), Pearson code *oP*44 and Wyckoff sequence 33,*a* ^11^. See Table 1 for selected crystallographic data and details of the structure determinations. Atomic coordinates, equivalent isotropic and anisotropic displacement parameters are reported in the Supporting Information.

**Table 1:** Selected crystallographic data and details of the structure determinations of [ClOF_2_][*M*F_6_] (*M*=P, V, Ru, Ir, Os, Sb). Columns are arranged in order of increasing *r*_eff_(*M* ^*V*^).

Compound	[ClOF_2_][PF_6_]	[ClOF_2_][VF_6_]	[ClOF_2_][RuF_6_]	[ClOF_2_][IrF_6_]	[ClOF_2_][OsF_6_]	[ClOF_2_][SbF_6_]
molar mass [g⋅mol^−1^]	234.42	254.39	304.52	395.65	393.65	325.20
space group (No.)	*Pna*2_1_ (33)					
*a* [Å]	14.322(3)	14.7272(9)	14.882(3)	14.7229(15)	15.0037(6)	15.1032(6)
*b* [Å]	5.1046(10)	5.1331(2)	5.1859(10)	5.1304(8)	5.2277(2)	5.2766(3)
*c* [Å]	7.9096(16)	8.1446(4)	8.2299(16)	8.1328(8)	8.2816(3)	8.2943(3)
*V* [Å^3^]	578.2(2)	615.70(5)	635.2(2)	614.31(13)	649.57(4)	661.00(5)
*Z*	4					
pearson symbol	*oP*44					
*ρ*_*calc*._ [g⋅cm^−3^]	2.69	2.74	3.19	4.28	4.03	3.27
*μ* [mm^−1^]	1.060	2.159	2.999	22.284	20.150	4.685
color	colorless	colorless	colorless	yellow	colorless	colorless
crystal morphology	block	block	block	plate	plate	plate
crystal size [mm^3^]	0.27⋅0.28⋅0.41	0.22⋅0.23⋅0.34	0.13⋅0.16⋅0.22	0.04⋅0.09⋅0.11	0.06⋅0.16⋅0.17	0.03⋅0.04⋅0.12
*T* [K]	100					
*λ* [Å]	0.71073 (Mo‐K_α_)					
no. of reflections	6097	10 498	8305	8014	9961	9139
*θ* range [°]	2.85–32.03	2.77–32.03	2.74–31.45	2.77–31.87	2.72–36.40	2.70–33.33
range of Miller indices	−21≤*h*≤21	−21≤*h*≤21	−21≤*h*≤21	−21≤*h*≤21	−25≤*h*≤24	−23≤*h*≤23
	−7≤*k*≤7	−7≤*k*≤7	−7≤*k*≤7	−7≤*k*≤7	−8≤*k*≤8	−8≤*k*≤8
	−11≤*l*≤11	−12≤*l*≤10	−12≤*l*≤12	−10≤*l*≤12	−13≤*l*≤13	−12≤*l*≤12
absorption correction	integration	integration	integration	integration	multi‐scan	integration
*Trans*._max_, *Trans*._min_	0.79, 0.71	0.70, 0.49	0.72, 0.62	0.50, 0.02	0.15, 0.07	0.91, 0.35
*R*_int_, *R*_*σ*_	0.0186, 0.0310	0.0290, 0.0495	0.0199, 0.0336	0.0112, 0.0193	0.0292, 0.0412	0.0153, 0.0250
completeness of the data set	1.000	0.997	1.000	0.990	0.998	0.999
no. of unique reflections	2016	2030	2113	1884	3124	2474
no. of parameters	101	101	101	102	101	101
no. of restraints	1	1	1	1	1	1
no. of constraints	0	0	0	0	0	0
*S* (all data)	1.07	1.08	1.10	1.17	0.95	1.10
*R*(*F*) (*I*≥2*σ*(*I*), all data)	0.0261, 0.0270	0.0281, 0.0339	0.0216, 0.0230	0.0191, 0.0211	0.0197, 0.0242	0.0230, 0.0253
*wR*(*F* ^*2*^) (*I*≥2*σ*(*I*), all data)	0.0685, 0.0691	0.0659, 0.0686	0.0542, 0.0548	0.0492, 0.0513	0.0402, 0.0417	0.0576, 0.0590
flack parameter *x*	0.29(9)	0.015(15)	−0.02(3)	0.136(16)	0.024(6)	0.22(3)
extinction coefficient	not refined	0.007(2)	0.0018(6)	0.0009(2)	0.0040(3)	not refined
Δ*ρ*_max_, Δ*ρ*_min_ [e⋅Å^−3^]	0.30, −0.58	0.45, −0.63	0.59, −0.63	1.11, −1.03	1.48, −1.60	1.51, −0.81

**Figure 1:**
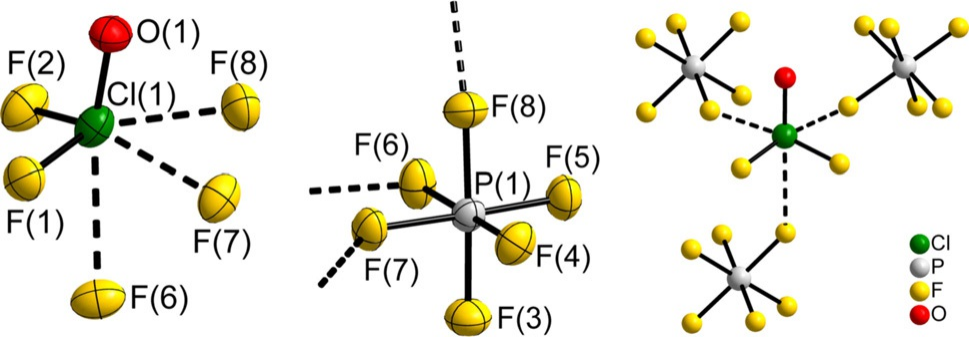
Cation and anion environments in the crystal structure of [ClOF_2_][PF_6_]. The difluorooxychloronium(V) hexafluorido(non)metallates(V), [ClOF_2_][*M*F_6_] (*M*=V, Ru, Os, Ir, P, As, Sb) are isotypic.^[38]^ The short contacts of the F atoms belonging to the [PF_6_]^−^ anions with the neighboring Cl atoms are shown as dashed bonds. On the left and in the middle, atoms are shown with displacement ellipsoids at the 70 % probability level at 100 K. On the right, atoms are shown as isotropic with arbitrary radii.

The chlorine atom Cl(1) occupies the Wyckoff position 4*a* (site symmetry 1/ *C*_1_) and is surrounded by the fluorine atoms F(1) and F(2) as well as the oxygen atom O(1), giving the trigonal pyramidal [ClOF_2_]^+^ cation, see Figure 1. Such a trigonal pyramidal geometry is also observed for the heavier homologues [BrOF_2_]^+^ in [BrOF_2_][AsF_6_] and [IOF_2_]^+^ in [IOF_2_][IO_2_F_4_] as well as in the crystal structures of the valence isoelectronic chalcogen oxyfluorides SOF_2_ and SeOF_2_.[^38^, ^50^, ^51^, ^52^]

The respective Cl−O and Cl−F bond lengths within the [ClOF_2_]^+^ cations of the investigated compounds, along with the As compound from the literature, are stated in Table 2. The Cl−O bond lengths range from 1.4364(17) Å in [ClOF_2_][PF_6_] to 1.477(4) Å in [ClOF_2_][OsF_6_] and are longer than in gaseous ClOF_3_ (1.405(3) Å, from electron diffraction at 238 K).^[53]^ A comparison with the crystal structure of ClOF_3_ is of limited use, because of O/F disorder, with averaged Cl−O/F bond lengths of 1.498(1) Å (data from single‐crystal X‐ray diffraction at 123 K) which is approximately the arithmetic mean of the Cl−O and Cl−F bond lengths from the electron‐diffraction study on gaseous ClOF_3_.^[31]^

**Table 2:** Experimental and calculated Cl−O and Cl−F bond lengths as well as Cl⋅⋅⋅F distances of the bridging F atoms of the fluoridometallate anions in [ClOF_2_][*M*F_6_] (*M*=V, Nb, Ta, Ru, Os, Ir, P, As, Sb). Rows are listed according to increasing *r*_eff_(*M* ^*V*^).

Compound	Exptl. bond lengths and distances [Å]			Calc. bond lengths and distances (DFT‐PBE0/TZVP, solid‐state) [Å]		
	Cl−O	Cl−F	Cl⋅⋅⋅F	Cl−O	Cl−F	Cl⋅⋅⋅F
[ClOF_2_][PF_6_]	1.4364(17)	1.5233(19), 1.5367(17)	2.4766(17)–2.6061(16)	1.41	1.62, 1.62	2.39–2.65
[ClOF_2_][AsF_6_]^[a]^	1.455(2)	1.522(2), 1.543(2)	2.476(2)–2.598(2)	1.41	1.62, 1.63	2.37–2.62
[ClOF_2_][VF_6_]	1.465(2)	1.528(2), 1.531(3)	2.362(2)–2.512(2)	1.41	1.62, 1.63	2.28–2.58
[ClOF_2_][RuF_6_]	1.461(3)	1.509(3), 1.533(3)	2.418(2)–2.522(3)	1.41	1.62, 1.63	2.33–2.58
[ClOF_2_][IrF_6_]	1.444(5)	1.486(6), 1.514(5)	2.401(5)–2.487(5)	1.41	1.63, 1.63	2.29–2.55
[ClOF_2_][OsF_6_]	1.477(4)	1.508(3), 1.532(4)	2.442(3)–2.520(3)	1.41	1.63, 1.63	2.36–2.52
[ClOF_2_][SbF_6_]	1.476(3)	1.497(3), 1.529(3)	2.458(3)–2.531(3)	1.41	1.62, 1.63	2.36–2.57
[ClOF_2_][NbF_6_]^[b]^	1.487(2)–1.511(2)	1.503(3)–1.516(2)	2.413(2)–2.484(2)	1.41, 1.41	1.63–1.64	2.30–2.55
[ClOF_2_][TaF_6_]^[b]^	1.488(4)–1.512(4)	1.488(4)–1.521(4)	2.423(4)–2.484(4)	1.41, 1.41	1.62–1.63	2.31–2.54

[a] Experimentally determined bond lengths taken from a previous study conducted at 100 K.^[38]^ [b] Experimentally determined bond lengths include Cl−O/F bond lengths of disordered [ClOF_2_]^+^ cations. Calculated bond lengths are given for a fully ordered, minimum‐energy model, see the Supporting Information.

The observed Cl−O bond lengths seem to correlate with the effective ionic radii of the *M* ^*V*^ atoms, *r*_eff_(*M* ^*V*^), within the [*M*F_6_]^−^ counter anions and seem to increase with increasing *r*_eff_(*M* ^*V*^). The effective ionic radii for *M* ^*V*^ atoms with coordination number six are: P: 0.38; As: 0.46; V: 0.54; Ru: 0.565; Ir: 0.57; Os: 0.575; Sb: 0.60 Å.^[54]^ Plots of the respective bond lengths and bond angles versus *r*_eff_(*M* ^*V*^) are given in the Supporting Information.

In contrast, no clear trend for the Cl−F bond lengths is observed, where the shortest value, 1.486(6) Å, is observed for [ClOF_2_][IrF_6_] and the longest values, 1.5367(17) Å, for [ClOF_2_][PF_6_]. However, for *M*=Os, which one would expect to be very similar to *M*=Ir, the observed Cl−F bond length is 1.508(3) Å. The aforementioned are shorter than the Cl−F bonds in gaseous ClOF_3_ with 1.603(4) Å (equatorial) and 1.713(3) Å (axial)) or in solid ClOF_3_ with 1.498(1) Å (equatorial, O/F disorder) and 1.683(2)–1.818(2) Å (axial), as would be expected.[^31^, ^53^]

One may attribute the elongation of the Cl−O bonds to interactions with the surrounding anions so that the coordination number of the Cl atom becomes 3+3 (see below) compared to gaseous ClOF_3_ where the coordination number is only four. However, this is in contradiction to the observed shortening of the Cl−F bonds, which also should be elongated due to the increased coordination number. For the [ClOF_2_]^+^ cation, one would expect shorter Cl−F and Cl−O bond lengths relative to ClOF_3_ due to the smaller coordination number of the Cl atom, less steric repulsion of ligands, and the positive charge of the cation. The expectation is in line with the observed Cl−F distances that are shorter compared to those of ClOF_3_, however, the observed Cl−O bond lengths contradict this expectation.

Correlations with *r*_eff_(*M* ^*V*^) are also observed for the F‐Cl‐O and F‐Cl‐F bond angles within the [ClOF_2_]^+^ cations, which are stated in Table 3. While the former decrease to some extent with increasing *r*_eff_(*M* ^*V*^), the latter increase.

**Table 3:** Selected experimental and calculated bond angles for [ClOF_2_][*M*F_6_] (*M*=V, Ru, Os, Ir, P, As, Sb). Rows are arranged in order of increasing *r*_eff_(*M* ^*V*^).

Compound	Exptl. bond angle [°]				Calc. bond angle (DFT‐PBE0/TZVP, solid‐state) [°]			
	F‐Cl‐F	F‐Cl‐O	F(6)⋅⋅⋅Cl‐O	F(7)⋅⋅⋅Cl⋅⋅⋅F(8)	F‐Cl‐F	F‐Cl‐O	F(6)⋅⋅⋅Cl‐O	F(7)⋅⋅⋅Cl⋅⋅⋅F(8)
[ClOF_2_][PF_6_]	98.00(10)	105.23(11), 105.46(11)	169.47(9)	79.99(6)	93.0	106.1, 106.9	175.2	85.7
[ClOF_2_][AsF_6_]^[a]^	98.7(1)	104.2(1), 105.5(1)	168.5(1)	88.48(10)	93.0	106.1, 106.5	174.0	85.9
[ClOF_2_][VF_6_]	98.53(14)	102.70(14), 104.83(14)	169.12(12)	84.42(8)	90.1	105.0, 105.6	174.0	90.3
[ClOF_2_][RuF_6_]	99.69(16)	103.51(16), 104.25(16)	167.33(14)	81.60(9)	92.5	105.7, 106.4	173.2	87.7
[ClOF_2_][IrF_6_]	100.0(3)	103.5(3), 104.1(3)	167.0(3)	80.36(17)	92.7	105.6, 106.4	171.9	84.1
[ClOF_2_][OsF_6_]	100.4(2)	103.3(2), 104.15(19)	166.84(17)	80.33(10)	92.4	106.0, 106.4	168.9	85.6
[ClOF_2_][SbF_6_]	100.66(19)	102.90(19), 104.57(18)	166.87(16)	79.85(10)	92.9	106.3, 106.4	172.1	85.2

[a] Experimental values are taken from a previous study conducted at 100 K.^[38]^

The *M* ^*V*^ atoms of the [*M*F_6_]^−^ anions occupy the general position 4*a* (site symmetry 1/*C*_1_). The observed *M*−F bond lengths are given in Table 4. Three of the six F atoms of the [*M*F_6_]^−^ anions—F(6), F(7) and F(8)—show *M–*μ‐F⋅⋅⋅Cl contacts with the Cl atoms of the [ClOF_2_]^+^ cation (Figure 1). These Cl⋅⋅⋅μ‐F distances lie in the range of approximately 2.4 to 2.6 Å within the series and are provided in Table 2. The corresponding *M*−F bond lengths of the bridging μ‐F atoms are thus longer than the non‐bridging, terminally bound F atoms (F(3), F(4) and F(5)). This is likely due to the higher effective coordination number of the bridging μ‐F atoms in their interactions with the Cl atoms.

**Table 4:** Experimental and calculated *M*‐F bond lengths of non‐bridging and bridging F atoms of the fluorido(non)metallate anions in [ClOF_2_][*M*F_6_] (*M*=V, Nb, Ta, Ru, Os, Ir, P, As, Sb). Rows are arranged in order of increasing *r*_eff_(*M* ^*V*^).

Compound	Exptl. bond length/Å		Calc. bond length (DFT‐PBE0/TZVP, solid‐state)/Å	
	*M*−F (non‐bridging)	*M*−F (bridging)	*M*−F (non‐bridging)	*M*−F (bridging)
[ClOF_2_][PF_6_]	1.5827(16)–1.5952(17)	1.6076(17)‐1.6255(16)‐	1.61–1.62	1.64–1.67
[ClOF_2_][AsF_6_]^[a]^	1.709(2)–1.7149(19)	1.7313(19)–1.7474(19)	1.71–1.72	1.74–1.77
[ClOF_2_][VF_6_]	1.734(2)–1.748(2)	1.794(2)–1.820(2)	1.72–1.74	1.78–1.85
[ClOF_2_][RuF_6_]	1.832(2)–1.836(2)	1.867(2)–1.875(2)	1.85–1.86	1.89–1.91
[ClOF_2_][IrF_6_]	1.828(5)–1.841(4)	1.856(4)–1.869(4)	1.83–1.87	1.89–1.94
[ClOF_2_][OsF_6_]	1.855(3)–1.866(3)	1.882(3)–1.894(3)	1.88–1.89	1.92–1.94
[ClOF_2_][SbF_6_]	1.857(3)‐1.863(3)	1.881(3)‐1.893(2)	1.90–1.91	1.93–1.95
[ClOF_2_][NbF_6_]	1.857(2)–1.867(2)	1.913(2)–1.926(2)	1.87–1.89	1.92–1.97
[ClOF_2_][TaF_6_]	1.863(4)–1.872(4)	1.911(3)–1.923(3)	1.88–1.89	1.93–1.97

[a] Experimental values are taken from a previous study conducted at 100 K.^[38]^

For the Cl atoms, an overall coordination number of six results with a coordination sphere that can be best described as distorted octahedral. If the lone‐pair of the Cl atom is included, an *AX*_6_ *E* VSEPR arrangement of ligand atom‐chlorine bond pairs and the valence electron pair on chlorine is obtained (*A*=Cl, *X*=ligand and *E*=lone pair). The VSEPR model predicts a distorted pentagonal‐bipyramidal geometry for an *AX*_6_ *E* species.[^55^, ^56^, ^57^] However, no species with such an arrangement of the ligands is known.^[58]^ Instead, two different structures of *AX*_6_ *E* species, octahedral or distorted octahedral, have been described.^[58]^ An octahedral structure is observed, when the central atom has a sterically inactive lone pair, such as in [BiCl_6_]^3−^ and [BrF_6_]^−^.[^59^, ^60^] A distorted octahedral structure is observed, when the central atom has a “weakly” sterically active lone pair, such as in [IF_6_]^−^ and XeF_6_.[^61^, ^62^, ^63^]

In the case of the Cl atoms studied in these systems, a clear classification is difficult, because the ligands are different and the primary bond lengths of the [ClOF_2_]^+^ cation differ significantly from the Cl⋅⋅⋅μ‐F distances. The lone pair of the Cl atom might be arranged as in the free [ClOF_2_]^+^ cation, which has a distorted tetrahedral structure (*AX*_3_ *E* VSEPR arrangement).^[3]^ It might then point in the direction of the center of the three μ‐F atoms, that is, to a wider face of the octahedron.

The Br atom in the homologous [BrOF_2_]^+^ cation of [BrOF_2_][AsF_6_] has a similar distorted octahedral coordination environment with bridging μ‐F atoms.^[38]^ In the compound [IOF_2_][IO_2_F_4_], the I atom of the [IOF_2_]^+^ cation also exhibits a distorted octahedral coordination environment, but with close contacts to μ‐O instead of μ‐F atoms.^[50]^

**Figure 2:**
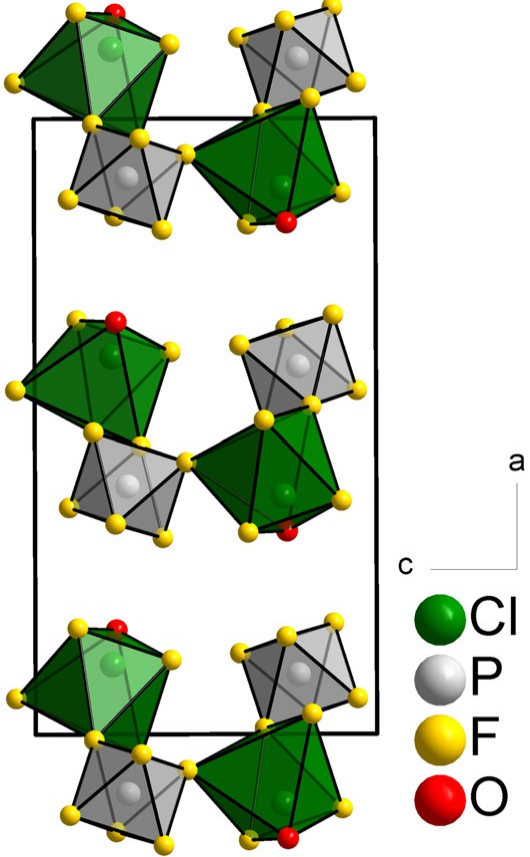
Crystal structure of [ClOF_2_][PF_6_] projected along the *b* axis. The difluorooxychloronium(V) hexafluorido(non)metallates(V), [ClOF_2_][*M*F_6_] (*M*=V, Ru, Os, Ir, P, As, Sb), are isotypic.^[38]^ The coordination polyhedra of the Cl atoms are shown in green and those of the P atoms in grey. Atoms are shown as isotropic with arbitrary radii.

In the compounds presented here, the bridging via μ‐F atoms originates from three [*M*F_6_]^−^ anions (Figure 1). When these interactions are considered, a layer motif, with corrugated layers parallel to the *bc* plane, is obtained (Figure 2). The crystal structures of the compounds can thus be described as layered structures with the Niggli formula ${}_{\infty }{}^{2}\left[\left\langle \mathrm{Cl}O_{\frac{1}{1}}F_{\frac{2}{1}}F_{\frac{3}{1+1}}\right\rangle \left\langle MF_{\frac{3}{1}}F_{\frac{3}{1+1}}\right\rangle \right]$ (*M*=V, Ru, Os, Ir, P, As, Sb), where the notation of the Niggli formula is given according to the literature.^[64]^

### Crystal structures of [ClOF_2_][*M*F_6_] (*M*=Nb, Ta)

The crystal structures of [ClOF_2_][*M*F_6_] (*M*=Nb, Ta) were determined by single‐crystal X‐ray diffraction. Both crystallize with eight formula units per unit cell in the orthorhombic space group *Pna*2_1_ (No. 33), Pearson code *oP*88 and Wyckoff sequence 33,*a* ^22^. See Table 5 for selected crystallographic data and details of the structure determinations. Atomic coordinates, equivalent isotropic and anisotropic displacement parameters are reported in the Supporting Information.

**Table 5:** Selected crystallographic data and details of the structure determinations of [ClOF_2_][*M*F_6_] (*M*=Nb, Ta).

Compound	[ClOF_2_][NbF_6_]	[ClOF_2_][TaF_6_]
molar mass [g⋅mol^−1^]	296.36	384.40
space group (No.)	*Pna*2_1_ (33)	
*a* [Å]	30.1890(11)	30.2598(16)
*b* [Å]	5.2653(2)	5.2923(3)
*c* [Å]	8.3588(3)	8.3610(4)
*V* [Å^3^]	1328.67(8)	1338.96(12)
*Z*	8	
Pearson symbol	*oP*88	
*ρ*_*calc*._ [g⋅cm^−3^]	2.96	3.81
*μ* [mm^−1^]	2.320	16.922
color	colorless	colorless
crystal morphology	block	block
Crystal size [mm^3^]	0.11⋅0.15⋅0.17	0.07⋅0.10⋅0.10
*T* [K]	100	
*λ* [Å]	0.71073 (Mo‐K_α_)	
no. of reflections	31 305	21 295
*θ* range [°]	2.70–34.92	2.69–33.21
range of Miller indices	−48≤*h*≤47	−46≤*h*≤46
	−8≤*k*≤8	−7≤*k*≤8
	−13≤*l*≤13	−12≤*l*≤12
absorption correction	multi‐scan	multi‐scan
*Trans*._max_, *Trans*._min_	0.75, 0.68	0.30, 0.12
*R*_int_, *R*_*σ*_	0.0263, 0.0311	0.0313, 0.0313
completeness of the data set	0.998	0.996
no. of unique reflections	5802	4982
no. of parameters	203	206
no. of restraints	2	3
no. of constraints	0	0
*S* (all data)	1.16	1.06
*R*(*F*) (*I*≥2*σ*(*I*), all data)	0.0293, 0.0356	0.0210, 0.0252
*wR*(*F* ^*2*^) (*I*≥2*σ*(*I*), all data)	0.0468, 0.0482	0.0417, 0.0432
flack parameter *x*	0.013(11)	0.074(11)
extinction coefficient	0.00299(15)	0.00142(9)
Δ*ρ*_max_, Δ*ρ*_min_ [e⋅Å^−3^]	1.08, −0.83	1.58, −1.83

The crystal structures of [ClOF_2_][NbF_6_] and [ClOF_2_][TaF_6_] are closely related to those described above, because the *a* axes of the former are approximately doubled. For example, *a=*30.1890(11) Å for [ClOF_2_][NbF_6_], whereas in [ClOF_2_][SbF_6_] the *a* axis is 15.1032(6) Å long, resulting in a different stacking of layers.^[38]^

**Figure 3:**
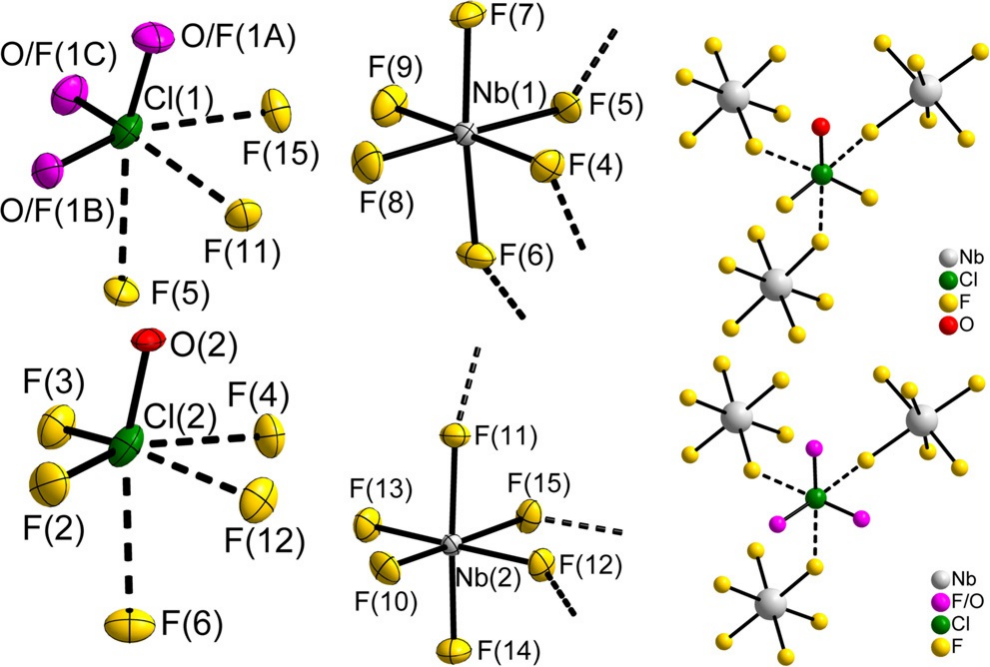
Cation and anion environments in the crystal structure of [ClOF_2_][NbF_6_]. The site occupancy factors for the O/F disordered [Cl(1)OF_2_]^+^ cation are: O/F(1A): 0.26(3) O/ 0.74 F; O/F(1B): 0.37(3) O/ 0.63 F; O/F(1C): 0.37(3) O/ 0.63 F. The close contacts of μ‐F atoms belonging to the [NbF_6_]^−^ anions with neighboring Cl atoms are shown as dashed bonds. On the left and in the middle, the atoms are shown with displacement ellipsoids at the 70 % probability level at 100 K. On the right, atoms are shown isotropic with arbitrary radii.

There are two symmetry‐independent Cl atoms present, Cl(1) and Cl(2), which both occupy the general 4*a* position with site symmetry 1/*C*_1_. In case of [ClOF_2_][NbF_6_], the [Cl(1)OF_2_]^+^ cation shows O/F disorder (Figure 3), where the Cl−O/F bond lengths of 1.501(2) to 1.511(2) Å are equal within the tripled standard uncertainties.

For the homologous [BrOF_2_]^+^ cation in [BrOF_2_][AsF_6_], which crystallizes in the cubic space group *P*2_1_3 (No. 198), O/F disorder was also observed.^[38]^ Furthermore, O/F disorder was observed in the crystal structure of ClOF_3_, as mentioned above. The Cl−O/F bond lengths of 1.498(1) Å (from single‐crystal X‐ray diffraction at 123 K) are close to the present reported values.^[31]^ In contrast, the second cation, [Cl(2)OF_2_]^+^, does not show O/F disorder, because the Cl−O bond length of 1.487(2) Å is significantly shorter than the Cl−F bond lengths of 1.514(2) and 1.516(2) Å.

**Figure 4:**
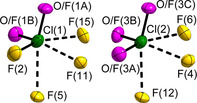
Cation environments in the crystal structure of [ClOF_2_][TaF_6_]. The site occupancy factors for the O/F disordered [Cl(1)OF_2_]^+^ cation are: O/F(1A): 0.58(3) O/ 0.42 F; O/F(1B): 0.42(3) O/ 0.58 F. The site occupancy factors for the O/F disordered [Cl(2)OF_2_]^+^ cation are: O/F(3A): 0.33(4) O/ 0.67 F; O/F(3B): 0.38(4) O/ 0.62 F; O/F(3C): 0.29(4) O/ 0.71 F. The same numbering Scheme of the F atoms in the [NbF_6_]^−^ anions in Figure 3 has been used for the F atoms in the [TaF_6_]^−^ anions of [ClOF_2_][TaF_6_]. The close contacts of μ‐F atoms belonging to the [TaF_6_]^−^ anions with neighboring Cl atoms are shown as dashed bonds. The displacement ellipsoids are shown at the 70 % probability level at 100 K.

The [ClOF_2_]^+^ cations in [ClOF_2_][TaF_6_] are either partially or fully O/F disordered (Figure 4). The [Cl(1)OF_2_]^+^ cation is partially O/F disordered with Cl−O/F bond lengths of 1.488(4) and 1.498(4) Å in comparison with the Cl−F bond length of 1.521(4) Å, whereas the [Cl(2)OF_2_]^+^ cation is fully disordered with Cl−O/F bond lengths of 1.493(4) to 1.512(4) Å.

It is unclear why both cations of the Ta compound show disorder. The chemical properties and effective ionic radii of Nb and Ta are similar, as are the [ClOF_2_]^+^ cation environments. The ordering of the cations may depend on temperature and cooling rate of the crystals, however the rate with which they were cooled for the diffraction experiment should be quite similar.

These observed Cl−O and Cl−F bond lengths nicely agree with the above‐mentioned correlation of bond lengths with the effective ionic radii *r*_eff_(*M* ^*V*^) in the compounds [ClOF_2_][*M*F_6_] (*M*=V, Ru, Os, Ir, P, As, Sb), as the former are increasing and the latter are decreasing with increasing *r*_eff_(*M* ^*V*^). Both Nb^V^ and Ta^V^ have an effective radius of 0.64 Å in coordination number six, which seemingly renders them to a border case, where O/F disorder can be observed.^[54]^ In contrast, O/F disorder is neither observed for [ClOF_2_][OsF_6_] (*r*_eff_(Os^V^): 0.575 Å) nor for [ClOF_2_][SbF_6_] (*r*_eff_(Sb^V^): 0.60 Å), see above.^[54]^

A similar trend is also observed for the bond angles within the [ClOF_2_]^+^ cations. The O/F−Cl(1)−O/F bond angles in the Nb compound lie in the range of 102.03(14) to 102.75(14)°, whereas the F−Cl(2)−O bond angles with 102.29(14) and 103.57(15)° and the F−Cl(2)−F bond angle of 100.95(15)° are significantly different. In the Ta compound, the respective O/F−Cl(1)−O/F bond angles are observed from 101.9(2) to 103.3(3)° and the O/F−Cl(2)−O/F bond angles from 101.3 to 103.3°.

**Figure 5:**
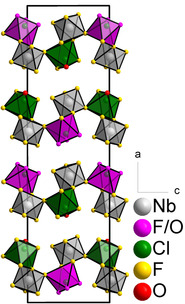
Crystal structure of [ClOF_2_][NbF_6_] projected along the *b* axis. The coordination polyhedra of the Cl(1) atoms (O/F disordered [ClOF_2_]^+^ cation) are shown in pink, that of the Cl(2) atoms (ordered [ClOF_2_]^+^ cation) in green and of the Nb atoms in grey. Atoms are shown as isotropic with arbitrary radii.

In the crystal structures of both compounds, two symmetry‐independent *M* ^*V*^ ions of respective [*M*F_6_]^−^ anions are present, which both occupy general 4*a* positions. As in the crystal structures of the compounds reported above, three of the F atoms of the [*M*F_6_]^−^ anions show close contacts to the Cl atoms of the [ClOF_2_]^+^ cations, see Figure 3. Thus, the F atoms act in a bridging manner *M*‐μ‐F⋅⋅⋅Cl. The coordination sphere of the Cl atoms is thus pseudo‐octahedral, as above. The respective Cl⋅⋅⋅μ‐F distances range from 2.413(2) to 2.484(2) Å in [ClOF_2_][NbF_6_] and from 2.423(4) to 2.484(4) Å in [ClOF_2_][TaF_6_]. The distances are shorter than in [ClOF_2_][PF_6_] or [ClOF_2_][RuF_6_] for example, see Table 2. The *M*‐F bond lengths of the non‐bridging, terminally bound F atoms, that is those not coordinating to Cl atoms, are shorter than the ones to the bridging μ‐F atoms, as expected (see Table 4). This observation can likely be explained by the higher effective coordination number of these bridging μ‐F atoms and their interaction with the Cl atoms. Similarly as in [ClOF_2_][*M*F_6_] (*M*=V, Ru, Os, Ir, P, As, Sb), a layer motif with corrugated layers parallel to the *bc* plane is obtained when the *M*‐μ‐F⋅⋅⋅Cl interactions are taken into account, see Figure 5, and the crystal structure of the compounds can be described as a layer structure with the same Niggli formula as above. In contrast, every second layer is shifted (and rotated) which leads to the approximate doubling of the *a* axis. So, the Nb and Ta compound crystallize in a stacking variant of likely very similar lattice energy and therefore even more modifications, that is, more stacking variants of these compounds could be obtainable.

### Quantum chemical calculations

The crystal structures of all thus far reported and herein described [ClOF_2_][*M*F_6_] compounds were investigated by means of quantum chemical solid‐state calculations with CRYSTAL17 (DFT‐PBE0/TZVP level of theory).[^65^, ^66^] The optimized structures are reported in the Supporting Information and comparisons of observed and calculated bond lengths and angles are given in Tables 2 and 3.

For [ClOF_2_][*M*F_6_] (*M*=Nb, Ta), several structural models with fully ordered [ClOF_2_]^+^ cations were optimized, that is, with full oxygen atom occupation on the possible positions. The structures of the respective models are reported in the Supporting Information. The energy differences of the two possible structural models of [ClOF_2_][NbF_6_] in comparison with the lowest‐energy model with full O atom occupation on the O/F(1C) position (Figure 3) are only ca. 6.0 and 6.6 kJ mol^−1^. For [ClOF_2_][TaF_6_], the energy differences lie in the range 4.7 to 9.6 kJ mol^−1^ with respect to the lowest‐energy model with full O atom occupation on the O/F(1B) and O/F(3B) positions. See Figure 4 and model No. 5 in the Supporting Information.

Surprisingly, the calculated Cl−O bond lengths (ca. 1.41 Å) are similar for all compounds investigated in this study. The Cl−O interaction is somewhat overestimated by the employed method, giving calculated bond lengths that are 2 to 7 % shorter than the observed values. The opposite trend is observed for the calculated Cl−F bond lengths, which are 5 to 9 % longer than the experimentally determined values and thus the interaction is underestimated. The respective calculated values for the [ClOF_2_]^+^ cations are overall independent of the [*M*F_6_]^−^ anion. Thus, these results are not in accordance with the crystal structures. In contrast, the calculated Cl⋅⋅⋅F distances overall correlate with *r*_eff_(*M* ^*V*^) and tend to decrease with increasing *r*_eff_(*M* ^*V*^). This is therefore in agreement with the experimental observations.

To get a qualitative picture of the bonding in these compounds, the atomic partial charges and overlap populations between atoms were examined by Mulliken population analyses. The average atomic partial charges are reported in Table 6 and the average overlap populations in Table 7. As for the calculated primary Cl−O and Cl−F bond lengths of the [ClOF_2_]^+^ cations, the average partial charges and average overlap populations are similar among the investigated compounds. The Cl−F interaction is rather ionic in comparison with the Cl−O interaction. The interactions between the Cl atoms and the bridging μ‐F atoms are by comparison also ionic. The values for the non‐bridging and bridging F atoms are likely dependent on both the electronegativity (*χ*_AR_) of *M* ^*V*^ and *r*_eff_(*M* ^*V*^).[^67^, ^68^] The average partial charges of *M* ^*V*^, F(non‐bridging) and μ‐F atoms show correlations with *χ*_AR_ that are smaller, the higher *χ*_AR_(*M*) is. Furthermore, the bridging μ‐F atoms have more negative partial charges than the non‐bridging F atoms, which is likely due to their higher coordination number.

**Table 6:** Average atomic partial charges from Mulliken population analyses of the optimized solid‐state structures of [ClOF_2_][*M*F_6_] (*M*=V, Nb, Ta, Ru, Os, Ir, P, As, Sb). Rows are arranged in order of increasing *r*_eff_(*M* ^*V*^).

Compound	Average atomic partial charge [*e*]					
	*M* ^*V*^	Cl	O	F (of [ClOF_2_]^+^)	F (non‐bridging)	μ‐F (bridging)
[ClOF_2_][PF_6_]	+1.80	+1.40	−0.18	−0.20	−0.42	−0.46
[ClOF_2_][AsF_6_]	+1.62	+1.39	−0.18	−0.20	−0.38	−0.42
[ClOF_2_][VF_6_]	+1.82	+1.41	−0.20	−0.22	−0.39	−0.47
[ClOF_2_][RuF_6_]	+1.96	+1.40	−0.19	−0.21	−0.42	−0.49
[ClOF_2_][IrF_6_]	+1.68	+1.39	−0.19	−0.21	−0.38	−0.44
[ClOF_2_][OsF_6_]	+1.84	+1.41	−0.18	−0.22	−0.41	−0.46
[ClOF_2_][SbF_6_]	+2.14	+1.40	−0.18	−0.21	−0.47	−0.51
[ClOF_2_][NbF_6_]^[a]^	+2.36	+1.42	−0.20	−0.22	−0.49	−0.55
[ClOF_2_][TaF_6_]^[a]^	+2.50	+1.42	−0.19	−0.22	−0.53	−0.57

[a] Values for a fully ordered model (without O/F disorder); see the Supporting Information for further details.

**Table 7:** Average overlap population between two atoms from Mulliken population analyses of the optimized solid‐state structures of [ClOF_2_][*M*F_6_] (*M*=V, Nb, Ta, Ru, Os, Ir, P, As, Sb). Rows are arranged in order of increasing *r*_eff_(*M* ^*V*^).

Compound	Average overlap population [*e*]				
	Cl−O	Cl−F	Cl⋅⋅⋅μ‐F	*M*−F (non‐bridging)	*M*‐μ‐F (bridging)
[ClOF_2_][PF_6_]	0.25	0.01	0.01	0.25	0.20
[ClOF_2_][AsF_6_]	0.24	0.01	0.01	0.28	0.24
[ClOF_2_][VF_6_]	0.25	0.01	0.01	0.11	0.10
[ClOF_2_][RuF_6_]	0.25	0.01	0.01	0.09	0.07
[ClOF_2_][IrF_6_]	0.24	0.01	0.01	0.12	0.11
[ClOF_2_][OsF_6_]	0.24	0.01	0.01	0.09	0.08
[ClOF_2_][SbF_6_]	0.25	0.01	0.01	0.19	0.16
[ClOF_2_][NbF_6_]^[a]^	0.25	0.01	0.01	0.10	0.08
[ClOF_2_][TaF_6_]^[a]^	0.25	0.01	0.01	0.12	0.10

[a] Values for a fully ordered model (without O/F disorder); see the Supporting Information for further details.

**Figure 6:**
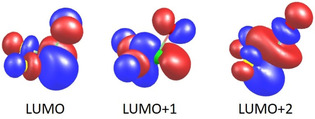
The three lowest unoccupied orbitals of [ClOF_2_]^+^. They are all anti‐bonding with respect to the Cl−O bond and the Cl−F bonds. Contours for amplitudes are drawn at ±0.05 atomic units.

We note that the experimentally observed trend of increasing Cl−O bond lengths in [ClOF_2_]^+^ with increasing effective radius of the central *M* atom in the [*M*F_6_]^−^ anions is not reproduced in the calculations, where a value of 1.41 Å is obtained, independent from the choice of *M*. Moreover, the entire electronic structure of the cation does not show a significant dependence on *M*, as reflected by the almost identical numbers for the partial charges and the overlap populations in [ClOF_2_]^+^ for all choices of *M* in Tables 6 and 7. For additional insights, we carried out molecular DFT calculations using TURBOMOLE and the same settings as for the periodic calculations for a bare [ClOF_2_]^+^ cation as well as for [[ClOF_2_][*M*F_6_]_3_]^2−^ anions (*M*=P, Sb). For the latter, the atomic coordinates of the three [*M*F_6_]^−^ ions were fixed to those of the determined crystal structures. Like for the periodic treatment reported above, Mulliken analyses indicate charge being transferred from the anion to the cation, here amounting to 0.28 electrons for both types of [*M*F_6_]^−^ anions. For the periodic treatment it is somewhat smaller, circa 0.2 electrons, see Table 6. This leads to partial occupations of the energetically lowest unoccupied orbitals of the bare [ClOF_2_]^+^ ion. The three energetically lowest of them are shown in Figure 6. These are the three energetically highest of the 16 molecular orbitals that may be constructed from the atomic valence s/p orbitals while the other thirteen molecular orbitals are occupied by the 26 valence electrons of the [ClOF_2_]^+^ cation. The three are all anti‐bonding with respect to the Cl−O bond as well as to the Cl−F bonds. Consequently, both Cl−O and Cl−F bonds are longer in the embedded than in the bare cation. For *M*=P they amount to 1.384 Å (Cl−O) and 1.596 Å (mean value of the two Cl−F bond lengths), thus longer by 0.005 Å and 0.039 Å than for the bare [ClOF_2_]^+^ ion. On the other hand, the changes from *M*=P to *M*=As are small, +0.002 Å for Cl−O and +0.003 Å for Cl−F, as the amount of charge transferred is the same for both cases. So, whereas changes from the bare to the embedded cation as well as the weak dependence of the Cl−F bond lengths on the choice of *M* result from our calculations and can be rationalized within a simple orbital picture, this is not the case for the correlations of Cl−O distances with any property of *M*.

### Raman spectra of [ClOF_2_][*M*F_6_] compounds

Compounds containing the [ClOF_2_]^+^ cation and a range of different anions, including “[F(HF)_*n*_]^−^”, [BF_4_]^−^, [MoOF_5_]^−^, [Mo_2_O_2_F_9_]^−^, [*M*F_6_]^−^ (*M*=V, Nb, Ta, U, Pt, Au, P, As, Sb, Bi) and [SiF_6_]^2−^, were previously extensively studied by Raman spectroscopy in hydrogen fluoride solutions and/or in the solid‐state.[^3^, ^20^, ^21^, ^33^, ^34^, ^35^, ^36^, ^37^, ^38^, ^69^, ^70^, ^71^] In the previous studies, the band assignments were usually based on a *C*_s_ symmetry for the [ClOF_2_]^+^ cation either in solution or in the solid‐state.

**Figure 7:**
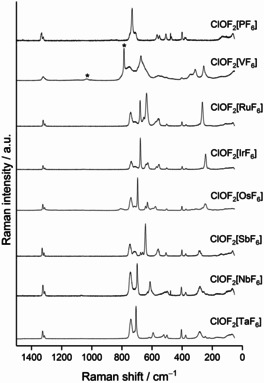
Recorded high‐resolution Raman spectra of solid [ClOF_2_][*M*F_6_] (*M*=V, Nb, Ta, Ru, Os, Ir, P, Sb). Asterisks denote bands that likely arise from hydrolysis products of the sample. Spectra are arranged in order of increasing *r*_eff_(*M* ^*V*^).

The Raman spectra of the presently investigated compounds are shown in Figure 7 and the respective low‐resolution spectra are reported in the Supporting Information. Additionally, Raman spectra based on the optimized solid‐state structures (DFT‐PBE0/TZVP level of theory) were calculated. A comparison of the calculated and experimental Raman spectra, along with band assignments for the calculated Raman spectra, are given in the Supporting Information. Vibrational frequencies attributable to the [ClOF_2_]^+^ cation are listed in Table 8. The Raman spectra of the compounds [ClOF_2_][*M*F_6_] (*M*=V, Nb, Ta, P, Sb) reported in this work nicely agree with the previously reported spectra.^[34]^

**Table 8:** Comparison of experimentally determined and calculated vibrational frequencies assigned to the [ClOF_2_]^+^ cation in [ClOF_2_][*M*F_6_] (*M*=V, Nb, Ta, Ru, Os, Ir, P, Sb). Rows are arranged in order of increasing *r*_eff_(*M* ^*V*^).

Compound	*v*(ClO) [cm^−1^]	*v*_s_(ClF_2_) [cm^−1^]	*v*_as_(ClF_2_) [cm^−1^]	*δ*_umbrella_(ClOF_2_) [cm^−1^]	*δ*_sciss_(ClO) [cm^−1^]	*δ*_sciss_(ClF_2_) [cm^−1^]	Reference
[ClOF_2_][PF_6_]	1332, 1321	732	710	508	401	382	this work
	1329–1327	748–742	727–715	495–486	388–386	352–350	calculated
	1334, 1321	741	710	506	400	382, 377	^[34]^
	1334, 1325	740	713	511	403	380	^[33]^
[ClOF_2_][VF_6_]	1322	750	712	506	401	345	this work
	1312–1310	737	718–702	487	401–399	361–343	calculated
	1318, 1308	746	not observed	530	407	380	^[34]^
[ClOF_2_][RuF_6_]	1323, 1311	742	714	503	401	375	this work
	1317–1315	745	715	491–481	392–390	350–349	calculated
[ClOF_2_][IrF_6_]	1324, 1313	737	713	503	400	374	this work
	1319–1317	743–737	727–708	491–481	390–384	349	calculated
[ClOF_2_][OsF_6_]	1325, 1313	740	716	503	402	375	this work
	1329–1323	757–735	699–692	486–476	386	348–347	calculated
[ClOF_2_][SbF_6_]	1330, 1318	747	715	507	402	377	this work
	1325–1324	751–743	727–714	494–484	389–388	348–347	calculated
	1327, 1316	742	715	508	402	377	^[34]^
	1330, 1319	748	709	509	404	378	^[20]^
[ClOF_2_][NbF_6_]	1325, 1313	744	not resolved	499	405	377	this work
	1317–1313	747–745	738–705	493–490	398–393	351–347	calculated
	1332, 1320	740	not observed	502	406	377	^[34]^
[ClOF_2_][TaF_6_]	1327, 1315	741	not resolved	500	404	376	this work
	1330–1316	765–741	722–711	500–468	395–388	350–349	calculated
	1324, 1313	737	not observed	502	406	377	^[34]^

Generally, six bands are observed and attributed to the [ClOF_2_]^+^ cation in most Raman spectra: *v*(ClO) at ≈1330 cm^−1^ (usually two separate bands due to the ^35^Cl and ^37^Cl isotopes), *v*_s_ (ClF_2_) at ≈740 cm^−1^, *v*_as_(ClF_2_) at ≈710 cm^−1^, *δ*_umbrella_([ClOF_2_]) at ≈510 cm^−1^, *δ*_sciss_(ClO) at ≈400 cm^−1^ and *δ*_sciss_(ClF_2_) at ≈380 cm^−1^.[^3^, ^35^] The frequencies that are assigned to these bands are similar among the [ClOF_2_][*M*F_6_] compounds, indicating that the [*M*F_6_]^−^ anion has a minor effect. The bands attributable to *v*(ClO) and *v*(ClF) modes in the Raman spectrum of liquid ClOF_3_ occur at 1224 and 689 cm^−1^, respectively, and are shifted bathochromically when compared with the [ClOF_2_]^+^ cation, as may be expected.^[72]^

The calculated frequencies of [ClOF_2_][*M*F_6_] compounds usually lie lower in energy and the band assignments of the calculated frequencies show that the vibrational modes of the [*M*F_6_]^−^ anions are vibrationally coupled to modes of the [ClOF_2_]^+^ cation. This was also shown for the calculated Raman spectrum for the hypothetical molecular anion, [ClOF_2_][AsF_6_]_3_^2−^, which was used to model a section of the crystal structure of [ClOF_2_][AsF_6_].^[38]^

Bands assigned to the [*M*F_6_]^−^ anions are in agreement with those reported for their respective lithium salts Li[*M*F_6_] (*M*=V, Nb, Ta, Ru, Os, Ir, P, Sb).^[73]^

## Conclusions

The reactions of chlorine trifluoride with oxides or with metals and O_2_ under UV irradiation led to the formation of the compounds [ClOF_2_][*M*F_6_] (*M*=V, Nb, Ta, Ru, Os, Ir, P, Sb). The crystal structures determined by single‐crystal X‐ray diffraction show that two different structure types are formed as determined by different stacking variants among the layer structures. The sizes of the *M* ^*V*^ ions generally impact the primary Cl−O bond lengths of the [ClOF_2_]^+^ cations, which increase with increasing effective ionic radius *r*_eff_(*M* ^*V*^). Surprisingly, quantum‐chemical calculations at the present level of theory did not reproduce the observed values, with longer Cl−F and much shorter Cl−O bond lengths. The Cl−O Raman shifts also generally do not to follow this trend. The calculations show that the Cl−O bonds have essentially covalent character, whereas the Cl−F bonds have a significantly higher degree of ionic character. The interactions of the μ‐F atoms of the [*M*F_6_]^−^ anions that bridge to the Cl atoms are essentially electrostatic. Further investigations regarding the photochemical synthesis of new [ClOF_2_]^+^ compounds are ongoing.

## Experimental Section

**General**: Volatile materials were handled in a Monel metal Schlenk line, which was passivated with undiluted fluorine and/or chlorine trifluoride at various pressures before use. Moisture‐sensitive compounds were stored and handled in an Ar‐filled glove box (MBraun). Reaction vessels were made out of fluoropolymer (PFA) that were closed by stainless‐steel valves. All reactors were passivated with fluorine before use. Preparations were carried out in an atmosphere of dry and purified argon (5.0, Praxair). Chlorine trifluoride was stored over NaF to remove traces of HF. Photochemical syntheses were carried out in a homemade UV reactor, which was equipped with eight low‐pressure Hg lamps (OSRAM Puritec HNS S 11 W G23, main emission line 254 nm). ***Caution**! Fluorine, chlorine trifluoride, and difluorooxychloronium(V) compounds must be handled using appropriate protective gear with ready access to proper emergency treatment procedures in the event of contact. They are potent oxidative fluorinators that are only stable under the rigorously anhydrous conditions employed in the experimental procedures outlined in this section. They can react vigorously to explosively upon hydrolysis or contact with organic materials. The utmost precautions must be taken when disposing of these materials and their derivatives. The PFA reaction vessels occasionally became brittle after prolonged UV irradiation of the reaction mixtures due to stress cracking likely caused by highly reactive radicals formed in these reactions*.

The low yields are due to incomplete transfer of the products from the PFA tubes into the storage vessels. For [ClOF_2_][PF_6_] and [ClOF_2_][VF_6_], the yields are even lower due to the dissociation vapor pressures of these compounds at room temperature, which were reported to be approximately 4.7 and 3.3 mbar, respectively.^[34]^

**Syntheses**—**General**: A PFA reaction vessel was loaded with the solid starting material outside the glove box and attached to a stainless‐steel valve. The valve was then connected to the Monel metal Schlenk line, the reaction vessel was evacuated and an excess of ClF_3_ was condensed onto the solid at 77 K. The reaction vessel was then placed in a stainless‐steel Dewar vessel and allowed to warm to room temperature over a period of several hours. After that, the reaction vessel was placed inside the UV reactor and irradiated. In the case of Os and Ir, the reaction mixtures were then cooled to 77 K, the liquid nitrogen cooling removed, and then O_2_ was added to the reaction vessels. UV‐irradiation was started while the reaction mixture warmed to room temperature. All volatiles were then pumped off and the product was isolated in the glove box.

**[ClOF_2_][VF_6_]**: 45 mg V_2_O_5_ (0.25 mmol) was reacted with 0.42 g ClF_3_ (4.5 mmol) and the reaction mixture was then irradiated for five days (30.8 mg isolated, 127 mg calculated).

**[ClOF_2_][NbF_6_]**: 27 mg Nb_2_O_5_ (0.10 mmol) was reacted with 0.36 g ClF_3_ (3.9 mmol) and the reaction mixture was then irradiated for four days (31.4 mg isolated, 60 mg calculated).

**[ClOF_2_][TaF_6_]**: 64 mg Ta_2_O_5_ (0.14 mmol) was reacted with 0.38 g ClF_3_ (4.1 mmol) and the reaction mixture was then irradiated for seven days (100 mg isolated, 108 mg calculated).

**[ClOF_2_][RuF_6_]**: 18 mg RuO_2_⋅*x* H_2_O (59.78 % Ru, *x* ≈2, 0.11 mmol) was reacted with 0.34 g ClF_3_ (3.7 mmol) and the reaction mixture was then irradiated for thirteen days (28.4 mg isolated, 32 mg calculated).

**[ClOF_2_][OsF_6_]**: 37 mg Os powder (0.19 mmol) was reacted with 0.60 g ClF_3_ (6.5 mmol), the reddish solution was frozen with liquid nitrogen, the cooling was removed and 0.7 bar O_2_ was added to the reaction vessel. The reaction mixture was then irradiated for 16 h (55.6 mg isolated, 75 mg calculated).

**[ClOF_2_][IrF_6_]**: 20 mg Ir powder (0.10 mmol) was reacted with 0.17 g ClF_3_ (1.8 mmol), the red solution was frozen with liquid nitrogen, the cooling was removed and 1 bar O_2_ was added to the reaction vessel. The reaction mixture was then irradiated for seven days (55.6 mg isolated, 75 mg calculated).

**[ClOF_2_][PF_6_]**: 38 mg P_2_O_5_ (0.27 mmol) was reacted with 0.36 g ClF_3_ (3.9 mmol) and the reaction mixture was then irradiated for four days (66.7 mg isolated, 127 mg calculated).

**[ClOF_2_][SbF_6_]**: 18 mg Sb_2_O_4_ (0.058 mmol) was reacted with 0.14 g ClF_3_ (1.5 mmol) and the reaction mixture was then irradiated for thirteen days (29.1 mg isolated, 38 mg calculated).

**Single‐crystal X‐ray diffraction**: Crystals of the moisture‐sensitive compounds were selected under dried perfluorinated oil (Fomblin YR1800, Solvay, stored over molecular sieves, 3 Å) and mounted on a MiTeGen loop. Intensity data of suitable crystals were recorded with a D8 Quest diffractometer (Bruker), an IPDS2 diffractometer (STOE) or an IPDS2T diffractometer (STOE). The diffractometers were operated with monochromatized Mo‐K_α_ radiation (0.71073 Å), multi‐layered optics (D8 Quest), or a graphite monochromator (IPDS2/IPDS2T) and equipped with a PHOTON 100 CMOS detector (D8 Quest) or an image plate detector (IPDS2/IPDS2T). Evaluation, integration and reduction of the diffraction data was carried out with the APEX3 software suite (D8 Quest) or the X‐Area software suite (IPDS2/IPDS2T).[^74^, ^75^] The diffraction data was corrected for absorption utilizing the multi‐scan method of SADABS within the APEX3 software suite (D8 Quest) or the integration method with the modules X‐Shape and X‐Red32 of the X‐Area software suite (IPDS2/IPDS2T).[^76^, ^77^, ^78^] The structures were solved with dual‐space methods (SHELXT) and refined against *F* ^2^ (SHELXL).[^79^, ^80^] For the compounds [ClOF_2_][NbF_6_] and [ClOF_2_][TaF_6_] partial or full O/F disorder was observed for the [ClOF_2_]^+^ cations. The *xyz* and *U*_ij_ parameters of the disordered O/F atoms were restrained with the EXYZ and EADP commands in SHELXL. The site occupancy factors among the possible O/F positions were restrained by the SUMP command in SHELXL. For [ClOF_2_][NbF_6_] the site occupation factors for the O/F disordered [Cl(1)OF_2_]^+^ cation are the following: O/F(1A): 0.26(3) O/ 0.74 F; O/F(1B): 0.37(3) O/ 0.63 F; O/F(1C): 0.37(3) O/ 0.63 F. For [ClOF_2_][TaF_6_] the site occupancy factors for the O/F disordered [Cl(1)OF_2_]^+^ cation are the following: O/F(1A): 0.58(3) O/ 0.42 F; O/F(1B): 0.42(3) O/ 0.58 F. The site occupancy factors for the O/F disordered [Cl(2)OF_2_]^+^ cation are the following: O/F(3A): 0.33(4) O/ 0.67 F; O/F(3B): 0.38(4) O/ 0.62 F; O/F(3C): 0.29(4) O/ 0.71 F. Weak systematic absence violations were observed for the space group *Pna*2_1_ (No. 33) for all here investigated compounds. However, the intensities of the respective reflections were close to the tripled standard uncertainties. Solution and refinement of the structures in crystallographic subgroups resulted in correlations between atomic coordinates and non‐positive definite displacement parameters for some atoms. The crystal structures were consequently solved and refined in the reported space groups, which were also indicated by the Addsym feature of the program package PLATON when searching for additional symmetry within the subgroups.[^81^, ^82^] The locations of highest residual electron densities after the final refinement were the following: [ClOF_2_][VF_6_]: 0.44 Å from atom O(1), [ClOF_2_][NbF_6_]: 1.46 Å from atom F(10), [ClOF_2_][TaF_6_]: 1.30 Å from atom F(11), [ClOF_2_][RuF_6_]: 0.92 Å from atom Ru(1), [ClOF_2_][OsF_6_]: 0.70 Å from atom Os(1), [ClOF_2_][IrF_6_]: 1.03 Å from atom F(3), [ClOF_2_][PF_6_]: 0.51 Å from atom O(1), [ClOF_2_][SbF_6_]: 0.97 Å from atom Sb(1). Representations of the crystal structures were created with the Diamond software.^[83]^

Deposition Number(s) 2013082, 2013083, 2013084, 2013085, 2013086, 2013087, 2013088, and 2013089 contain the supplementary crystallographic data for this paper. These data are provided free of charge by the joint Cambridge Crystallographic Data Centre and Fachinformationszentrum Karlsruhe Access Structures service.

**Raman spectroscopy**: The Raman spectra were measured with a Monovista CRS+ confocal Raman microscope (Spectroscopy & Imaging GmbH) using a 532 nm solid‐state laser and either a 300 grooves mm^−1^ (low‐resolution mode, FWHM: <4.62 cm^−1^) or a 1800 grooves mm^−1^ (high‐resolution mode, FWHM: <0.368 cm^−1^) grating. Samples were either transferred into dried glass vessels or immersed in perfluorinated oil (Fomblin YR1800, Solvay, stored over molecular sieve 3 Å) on a microscope slide.

**Quantum‐chemical calculations**: Periodic quantum‐chemical calculations were carried out for the difluorooxychloronium(V) hexafluorido(non)metallates(V) with the PBE0 hybrid density functional theory method (DFT‐PBE0).[^65^, ^66^] Triple‐zeta‐valence+polarization (TZVP) level basis sets were applied for all atoms. Details of the basis sets that were employed are given in the Supporting Information. All calculations were carried out with the CRYSTAL17 program package.^[84]^ The crystal structure of [ClOF_2_][AsF_6_] was taken from a previous study.^[38]^ The reciprocal space was sampled with the following Monkhorst‐Pack‐type *k*‐point grids: [ClOF_2_][VF_6_]: 2×4×3, [ClOF_2_][NbF_6_]: 1×5×3, [ClOF_2_][TaF_6_]: 1×5×3, [ClOF_2_][RuF_6_]: 2×4×3, [ClOF_2_][OsF_6_]: 2×4×3, [ClOF_2_][IrF_6_]: 2×4×3, [ClOF_2_][PF_6_]: 2×4×3, [ClOF_2_][AsF_6_]: 2×4×3, [ClOF_2_][SbF_6_]: 2×4×3. For the evaluation of the Coulomb and exchange integrals (TOLINTEG), tight tolerance factors of 8, 8, 8, 8, 16 were used for all calculations. Both the atomic positions and lattice parameters were fully optimized within the constraints imposed by the space group symmetry. Default DFT integration grids and optimization convergence thresholds were applied in all calculations. Mulliken population analyses were carried out for all compounds. For the magnetic systems [ClOF_2_][RuF_6_], [ClOF_2_][OsF_6_] and [ClOF_2_][IrF_6_] a ferromagnetic ground state was employed. The resulting magnetic moments were 3.0 μB for the [RuF_6_]^−^ anion (2.3 μB contribution from Ru), 3.0 μB for the [OsF_6_]^−^ anion (2.4 μB contribution from Os) and 2.0 μB for the [IrF_6_]^−^ anion (1.4 μB contribution from Ir). The harmonic vibrational frequencies and Raman intensities were obtained through usage of the computational Scheme implemented in CRYSTAL17.[^85^, ^86^, ^87^] The Raman intensities were calculated for a polycrystalline powder sample (total isotropic intensity in arbitrary units). The Raman spectra were obtained by using a pseudo‐Voigt band profile (50:50 Lorentzian:Gaussian) and an FWHM of 8 cm^−1^. The Raman spectra were simulated taking the experimental setup (*T=*293.15 K, *λ*=532 nm) into account. The band assignments were carried out by visual inspection of the normal modes with the Jmol program package.^[88]^ Molecular DFT calculations were carried out with TURBOMOLE with the same settings as the periodic calculations, orbitals were visualized with Chemcraft.[^89^, ^90^]

## Conflict of interest

The authors declare no conflict of interest.

## Supporting information

As a service to our authors and readers, this journal provides supporting information supplied by the authors. Such materials are peer reviewed and may be re‐organized for online delivery, but are not copy‐edited or typeset. Technical support issues arising from supporting information (other than missing files) should be addressed to the authors.

Supplementary
