# Supplementary material for: Photochemistry with Chlorine Trifluoride: Syntheses and Characterization of Difluorooxychloronium(V) Hexafluorido(non)metallates(V), [ClOF2][MF6] (M=V, Nb, Ta, Ru, Os, Ir, P, Sb)
Source: Chemistry. 2020 Dec 22;27(7):2381–92. doi: 10.1002/chem.202003629 (PMC7898883; doi:10.1002/chem.202003629)
Supplement: Supplementary file 1 — Supplementary [file CHEM-27-2381-s001.pdf]

Chemistry–A European Journal

Supporting Information

Photochemistry with Chlorine Trifluoride: Syntheses and Characterization of Difluorooxychloronium(V) Hexafluorido(non)metallates(V), $[ClOF_2][MF_6]$ (M = V, Nb, Ta, Ru, Os, Ir, P, Sb)

Benjamin Scheibe,[a] Antti J. Karttunen,[b] Florian Weigend,[a] and Florian Kraus*[a]

Content

Selected crystallographic data of difluorooxychloronium(V) fluoridometallates(V) 2

Plot of distances and angles within the $ClOF_2^+$ cations versus effective ionic radii of *M*(V) ions 8

Basis set details for quantum-chemical solid-state calculations 10

Lattice parameters and atomic coordinates of optimized solid-state structures of $ClOF_2[MF_6]$ (*M* = V, Nb, Ta, Ru, Os, Ir, P, As, Sb) 15

Comparison of low-resolution Raman spectra of $ClOF_2[MF_6]$ compounds (*M* = V, Nb, Ta, Ru, Os, Ir, P, Sb) 26

Raman spectra and band assignments for the calculated Raman spectra of $ClOF_2[MF_6]$ (*M* = V, Nb, Ta, Ru, Os, Ir, P, Sb) 27

References 34

Selected crystallographic data of difluorooxychloronium(V) fluoridometallates(V)

Atomic coordinates and displacement parameters of $ClOF_2[VF_6]$

Table S1. Atomic coordinates and equivalent isotropic displacement parameters U_{iso} of $ClOF_2[VF_6]$ at 100 K.

Atom	Position	*x*	*y*	*z*	U_{iso} / Å^2
V(1)	4*a*	0.39808(3)	0.39826(10)	0.72959(6)	0.01298(13)
Cl(1)	4*a*	0.39306(5)	0.10775(18)	0.27759(10)	0.0214(2)
O(1)	4*a*	0.34166(16)	0.3505(4)	0.2598(3)	0.0162(5)
F(1)	4*a*	0.32893(15)	−0.0769(4)	0.3681(3)	0.0230(5)
F(2)	4*a*	0.38619(15)	−0.0129(5)	0.1061(3)	0.0225(4)
F(3)	4*a*	0.33378(16)	0.1219(4)	0.7681(3)	0.0247(5)
F(4)	4*a*	0.30957(15)	0.5402(4)	0.6181(3)	0.0212(4)
F(5)	4*a*	0.36418(16)	0.5413(4)	0.9130(3)	0.0227(4)
F(6)	4*a*	0.49528(15)	0.2533(4)	0.8281(3)	0.0200(4)
F(7)	4*a*	0.43909(15)	0.2503(5)	0.5405(2)	0.0202(4)
F(8)	4*a*	0.46842(15)	0.6740(4)	0.6835(3)	0.0207(4)

Table S2. Anisotropic displacement parameters of $ClOF_2[VF_6]$ at 100 K.

Atom	U_{11} / Å^2	U_{22} / Å^2	U_{33} / Å^2	U_{23} / Å^2	U_{13} / Å^2	U_{12} / Å^2
V(1)	0.0120(2)	0.0145(2)	0.0125(2)	−0.0008(2)	−0.0003(2)	−0.00022(17)
Cl(1)	0.0133(3)	0.0300(4)	0.0209(4)	−0.0033(3)	−0.0028(3)	0.0002(3)
O(1)	0.0164(10)	0.0148(9)	0.0173(13)	0.0006(8)	−0.0020(9)	0.0050(8)
F(1)	0.0179(10)	0.0250(11)	0.0260(12)	0.0041(8)	0.0018(9)	−0.0034(8)
F(2)	0.0235(11)	0.0275(11)	0.0163(10)	−0.0053(9)	−0.0031(8)	−0.0001(9)
F(3)	0.0239(10)	0.0199(9)	0.0303(14)	0.0016(9)	0.0028(9)	−0.0070(8)
F(4)	0.0152(9)	0.0260(10)	0.0224(11)	0.0027(8)	−0.0025(9)	0.0028(8)
F(5)	0.0242(11)	0.0266(11)	0.0173(10)	−0.0060(9)	0.0031(9)	0.0021(9)
F(6)	0.0210(10)	0.0226(9)	0.0165(9)	−0.0006(8)	−0.0037(8)	0.0069(7)
F(7)	0.0190(9)	0.0270(10)	0.0146(10)	−0.0075(9)	−0.0011(7)	0.0020(8)
F(8)	0.0206(9)	0.0187(8)	0.0228(10)	0.0016(8)	−0.0012(8)	−0.0064(7)

Atomic coordinates and displacement parameters of $ClOF_2[PF_6]$

Table S3. Atomic coordinates and equivalent isotropic displacement parameters U_{iso} of $ClOF_2[PF_6]$ at 100 K.

Atom	Position	*x*	*y*	*z*	U_{iso} / Å^2
P(1)	4*a*	0.40302(4)	0.38662(11)	0.72980(7)	0.01791(12)
Cl(1)	4*a*	0.38681(4)	0.11177(11)	0.27429(8)	0.02260(13)
O(1)	4*a*	0.33048(12)	0.3438(3)	0.2620(2)	0.0232(3)
F(1)	4*a*	0.32779(11)	−0.0840(3)	0.3761(2)	0.0252(3)
F(2)	4*a*	0.37925(11)	−0.0211(4)	0.1024(2)	0.0287(3)
F(3)	4*a*	0.34159(12)	0.1345(3)	0.7644(2)	0.0283(3)
F(4)	4*a*	0.31949(11)	0.5163(3)	0.6259(2)	0.0253(3)
F(5)	4*a*	0.36850(11)	0.5177(3)	0.9002(2)	0.0257(3)
F(6)	4*a*	0.49026(11)	0.2551(3)	0.8287(2)	0.0265(3)
F(7)	4*a*	0.44176(11)	0.2541(3)	0.55662(19)	0.0248(3)
F(8)	4*a*	0.46730(12)	0.6375(3)	0.6904(2)	0.0276(3)

Table S4. Anisotropic displacement parameters of $ClOF_2[PF_6]$ at 100 K.

Atom	U_{11} / Å^2	U_{22} / Å^2	U_{33} / Å^2	U_{23} / Å^2	U_{13} / Å^2	U_{12} / Å^2
P(1)	0.0161(2)	0.0201(2)	0.0175(2)	−0.0011(2)	−0.00051(19)	0.00024(16)
Cl(1)	0.0172(2)	0.0281(3)	0.0225(2)	−0.0006(2)	−0.00145(18)	−0.00076(16)
O(1)	0.0232(7)	0.0202(6)	0.0262(9)	−0.0006(7)	−0.0027(7)	0.0047(6)
F(1)	0.0223(6)	0.0268(6)	0.0266(7)	0.0037(6)	0.0014(6)	−0.0035(5)
F(2)	0.0276(7)	0.0375(9)	0.0209(7)	−0.0057(7)	−0.0023(6)	0.0040(6)
F(3)	0.0300(7)	0.0246(6)	0.0304(9)	0.0017(6)	0.0021(7)	−0.0064(5)
F(4)	0.0200(6)	0.0305(7)	0.0254(7)	0.0028(6)	−0.0018(5)	0.0034(5)
F(5)	0.0243(7)	0.0311(8)	0.0218(7)	−0.0058(6)	0.0015(6)	0.0031(6)
F(6)	0.0243(7)	0.0327(8)	0.0226(6)	−0.0031(6)	−0.0039(5)	0.0091(5)
F(7)	0.0220(6)	0.0321(7)	0.0203(6)	−0.0074(6)	−0.0003(5)	0.0018(5)
F(8)	0.0254(7)	0.0261(6)	0.0314(8)	−0.0012(6)	0.0010(6)	−0.0066(6)

Atomic coordinates and displacement parameters of $ClOF_2[RuF_6]$

Table S5. Atomic coordinates and equivalent isotropic displacement parameters U_{iso} of $ClOF_2[RuF_6]$ at 100 K.

Atom	Position	*x*	*y*	*z*	U_{iso} / Å^2
Ru(1)	4*a*	0.59850(2)	0.60283(4)	0.26989(5)	0.01684(9)
Cl(1)	4*a*	0.61128(7)	0.89502(19)	0.72628(13)	0.0241(2)
O(1)	4*a*	0.66233(18)	0.6553(5)	0.7395(3)	0.0202(6)
F(1)	4*a*	0.67455(18)	1.0741(5)	0.6320(3)	0.0273(5)
F(2)	4*a*	0.61831(17)	1.0147(6)	0.8930(3)	0.0273(5)
F(3)	4*a*	0.6672(2)	0.8886(4)	0.2290(4)	0.0276(6)
F(4)	4*a*	0.69248(16)	0.4554(5)	0.3805(3)	0.0244(5)
F(5)	4*a*	0.63578(18)	0.4594(5)	0.0779(3)	0.0256(5)
F(6)	4*a*	0.49976(17)	0.7507(5)	0.1653(3)	0.0242(5)
F(7)	4*a*	0.55784(17)	0.7529(5)	0.4637(3)	0.0242(5)
F(8)	4*a*	0.52723(17)	0.3154(5)	0.3152(3)	0.0247(5)

Table S6. Anisotropic displacement parameters of $ClOF_2[RuF_6]$ at 100 K.

Atom	U_{11} / Å^2	U_{22} / Å^2	U_{33} / Å^2	U_{23} / Å^2	U_{13} / Å^2	U_{12} / Å^2
Ru(1)	0.01711(12)	0.01689(12)	0.01652(12)	−0.00086(14)	−0.00027(11)	0.00002(7)
Cl(1)	0.0188(4)	0.0297(5)	0.0238(4)	−0.0011(3)	−0.0021(3)	−0.0005(3)
O(1)	0.0218(11)	0.0167(10)	0.0222(17)	−0.0017(9)	−0.0019(9)	0.0049(8)
F(1)	0.0222(12)	0.0292(12)	0.0304(14)	0.0045(9)	0.0014(9)	−0.0050(9)
F(2)	0.0282(11)	0.0317(13)	0.0221(11)	−0.0052(11)	−0.0029(9)	0.0006(10)
F(3)	0.0293(12)	0.0209(10)	0.0325(14)	0.0026(9)	0.0018(10)	−0.0083(8)
F(4)	0.0183(10)	0.0292(10)	0.0258(11)	0.0042(10)	−0.0030(9)	0.0048(9)
F(5)	0.0298(12)	0.0262(10)	0.0207(10)	−0.0062(9)	0.0043(9)	0.0020(10)
F(6)	0.0242(11)	0.0266(11)	0.0217(10)	−0.0011(9)	−0.0047(9)	0.0078(8)
F(7)	0.0269(11)	0.0280(11)	0.0177(10)	−0.0068(9)	0.0003(8)	0.0026(9)
F(8)	0.0248(11)	0.0222(10)	0.0270(11)	0.0017(9)	−0.0006(8)	−0.0074(9)

Atomic coordinates and displacement parameters of $ClOF_2[SbF_6]$

Table S7. Atomic coordinates and equivalent isotropic displacement parameters U_{iso} of $ClOF_2[SbF_6]$ at 100 K.

Atom	Position	*x*	*y*	*z*	U_{iso} / Å^2
Sb(1)	4*a*	0.59745(2)	0.60365(4)	0.26865(6)	0.01624(7)
Cl(1)	4*a*	0.61420(8)	0.8957(2)	0.73000(13)	0.0233(2)
O(1)	4*a*	0.6660(2)	0.6591(6)	0.7403(4)	0.0204(7)
F(1)	4*a*	0.6756(2)	1.0721(6)	0.6355(4)	0.0264(6)
F(2)	4*a*	0.6207(2)	1.0093(6)	0.8950(4)	0.0278(6)
F(3)	4*a*	0.6667(2)	0.8876(5)	0.2285(4)	0.0284(6)
F(4)	4*a*	0.69092(18)	0.4481(6)	0.3768(4)	0.0250(5)
F(5)	4*a*	0.6304(2)	0.4659(6)	0.0715(3)	0.0251(5)
F(6)	4*a*	0.4981(2)	0.7597(5)	0.1734(3)	0.0248(5)
F(7)	4*a*	0.5605(2)	0.7474(5)	0.4666(3)	0.0242(5)
F(8)	4*a*	0.52459(19)	0.3235(5)	0.3142(3)	0.0237(5)

Table S8. Anisotropic displacement parameters of $ClOF_2[SbF_6]$ at 100 K.

Atom	U_{11} / Å^2	U_{22} / Å^2	U_{33} / Å^2	U_{23} / Å^2	U_{13} / Å^2	U_{12} / Å^2
Sb(1)	0.01783(11)	0.01677(10)	0.01410(10)	−0.00120(15)	−0.00016(10)	0.00005(6)
Cl(1)	0.0195(4)	0.0285(5)	0.0218(5)	0.0000(3)	−0.0023(3)	−0.0011(3)
O(1)	0.0237(13)	0.0150(10)	0.023(2)	−0.0006(10)	−0.0031(10)	0.0047(9)
F(1)	0.0242(13)	0.0298(14)	0.0253(14)	0.0055(10)	0.0003(10)	−0.0038(10)
F(2)	0.0303(13)	0.0328(16)	0.0203(12)	−0.0046(12)	−0.0023(10)	0.0014(12)
F(3)	0.0311(15)	0.0230(12)	0.0311(16)	0.0022(10)	0.0030(11)	−0.0080(9)
F(4)	0.0191(12)	0.0308(13)	0.0252(13)	0.0031(11)	−0.0044(10)	0.0045(10)
F(5)	0.0317(14)	0.0266(13)	0.0170(11)	−0.0054(10)	0.0047(10)	0.0016(11)
F(6)	0.0281(13)	0.0255(13)	0.0208(11)	−0.0018(10)	−0.0047(10)	0.0094(10)
F(7)	0.0285(13)	0.0288(12)	0.0154(11)	−0.0067(10)	0.0007(9)	0.0012(10)
F(8)	0.0249(12)	0.0220(11)	0.0240(12)	0.0018(10)	0.0006(8)	−0.0067(10)

Atomic coordinates and displacement parameters of $ClOF_2[OsF_6]$

Table S9. Atomic coordinates and equivalent isotropic displacement parameters U_{iso} of $ClOF_2[OsF_6]$ at 100 K.

Atom	Position	*x*	*y*	*z*	U_{iso} / Å^2
Os(1)	4*a*	0.59708(2)	0.60243(3)	0.26912(4)	0.01137(5)
Cl(1)	4*a*	0.61312(9)	0.8971(3)	0.72704(14)	0.0188(2)
O(1)	4*a*	0.6641(2)	0.6561(7)	0.7398(4)	0.0153(7)
F(1)	4*a*	0.6759(2)	1.0728(7)	0.6321(4)	0.0232(7)
F(2)	4*a*	0.6202(2)	1.0142(7)	0.8930(4)	0.0221(6)
F(3)	4*a*	0.6662(3)	0.8896(6)	0.2288(4)	0.0237(7)
F(4)	4*a*	0.6924(2)	0.4525(6)	0.3787(4)	0.0197(6)
F(5)	4*a*	0.6335(2)	0.4615(7)	0.0734(3)	0.0205(6)
F(6)	4*a*	0.4976(2)	0.7532(6)	0.1664(3)	0.0198(6)
F(7)	4*a*	0.5580(2)	0.7496(6)	0.4657(3)	0.0190(6)
F(8)	4*a*	0.5264(2)	0.3139(6)	0.3143(3)	0.0193(6)

Table S10. Anisotropic displacement parameters of $ClOF_2[OsF_6]$ at 100 K.

Atom	U_{11} / Å^2	U_{22} / Å^2	U_{33} / Å^2	U_{23} / Å^2	U_{13} / Å^2	U_{12} / Å^2
Os(1)	0.00949(7)	0.01331(7)	0.01130(6)	−0.00101(15)	−0.00011(9)	0.00005(5)
Cl(1)	0.0113(5)	0.0256(6)	0.0195(4)	0.0001(4)	−0.0029(3)	−0.0009(4)
O(1)	0.0155(15)	0.0110(13)	0.0193(19)	−0.0013(10)	−0.0033(11)	0.0051(12)
F(1)	0.0130(15)	0.0294(19)	0.0272(16)	0.0049(12)	0.0026(11)	−0.0059(13)
F(2)	0.0211(16)	0.0270(18)	0.0182(13)	−0.0056(12)	−0.0038(11)	0.0015(15)
F(3)	0.0229(17)	0.0204(15)	0.0277(16)	0.0005(10)	0.0026(11)	−0.0064(12)
F(4)	0.0134(14)	0.0243(15)	0.0212(13)	0.0034(11)	−0.0032(10)	0.0032(12)
F(5)	0.0215(17)	0.0247(16)	0.0154(12)	−0.0055(11)	0.0042(11)	0.0024(13)
F(6)	0.0188(15)	0.0234(16)	0.0171(12)	−0.0007(10)	−0.0048(10)	0.0083(12)
F(7)	0.0172(15)	0.0254(16)	0.0143(11)	−0.0066(10)	−0.0002(9)	0.0009(12)
F(8)	0.0169(15)	0.0198(14)	0.0211(12)	−0.0008(10)	−0.0015(9)	−0.0040(14)

Atomic coordinates and displacement parameters of $ClOF_2[IrF_6]$

Table S11. Atomic coordinates and equivalent isotropic displacement parameters U_{iso} of $ClOF_2[IrF_6]$ at 100 K.

Atom	Position	*x*	*y*	*z*	U_{iso} / Å^2
Ir(1)	4*a*	0.40276(2)	0.39654(4)	0.73031(8)	0.01379(8)
Cl(1)	4*a*	0.38700(13)	0.1039(4)	0.2724(2)	0.0208(4)
O(1)	4*a*	0.3363(3)	0.3437(10)	0.2589(7)	0.0177(13)
F(1)	4*a*	0.3238(3)	−0.0734(10)	0.3676(7)	0.0230(10)
F(2)	4*a*	0.3799(3)	−0.0152(11)	0.1063(7)	0.0241(10)
F(3)	4*a*	0.3340(4)	0.1062(8)	0.7701(7)	0.0247(12)
F(4)	4*a*	0.3071(3)	0.5440(10)	0.6182(6)	0.0207(9)
F(5)	4*a*	0.3644(3)	0.5390(10)	0.9242(6)	0.0209(9)
F(6)	4*a*	0.5023(3)	0.2485(9)	0.8352(6)	0.0199(9)
F(7)	4*a*	0.4436(3)	0.2482(9)	0.5339(5)	0.0204(9)
F(8)	4*a*	0.4735(3)	0.6868(9)	0.6838(6)	0.0212(9)

Table S12. Anisotropic displacement parameters of $ClOF_2[IrF_6]$ at 100 K.

Atom	U_{11} / Å^2	U_{22} / Å^2	U_{33} / Å^2	U_{23} / Å^2	U_{13} / Å^2	U_{12} / Å^2
Ir(1)	0.01440(11)	0.01430(11)	0.01266(12)	−0.00086(19)	−0.00002(14)	0.00001(6)
Cl(1)	0.0157(6)	0.0265(8)	0.0201(10)	−0.0011(6)	−0.0023(5)	−0.0006(5)
O(1)	0.019(2)	0.0146(18)	0.019(4)	−0.0035(19)	−0.0030(18)	0.0059(16)
F(1)	0.018(2)	0.027(2)	0.024(3)	0.0038(18)	0.0032(18)	−0.0032(18)
F(2)	0.025(2)	0.030(3)	0.018(2)	−0.005(2)	−0.0037(18)	0.000(2)
F(3)	0.026(2)	0.0166(19)	0.031(4)	0.0014(16)	−0.0009(18)	−0.0084(15)
F(4)	0.0169(19)	0.025(2)	0.020(2)	0.0055(18)	−0.0043(17)	0.0066(17)
F(5)	0.027(2)	0.020(2)	0.015(2)	−0.0051(17)	0.0039(17)	0.0016(18)
F(6)	0.020(2)	0.022(2)	0.0171(19)	−0.0024(16)	−0.0046(16)	0.0099(16)
F(7)	0.023(2)	0.0246(19)	0.013(2)	−0.0064(18)	0.0019(16)	0.0026(18)
F(8)	0.0204(19)	0.0191(19)	0.024(2)	0.0006(16)	0.0009(15)	−0.0063(17)

Atomic coordinates and displacement parameters of $ClOF_2[NbF_6]$

Table S13. Atomic coordinates and equivalent isotropic displacement parameters U_{iso} of $ClOF_2[NbF_6]$ at 100 K.

Atom	Position	*x*	*y*	*z*	U_{iso} / Å^2
Nb(1)	4*a*	0.42532(2)	0.53321(5)	0.51021(4)	0.00988(6)
Nb(2)	4*a*	0.17377(2)	0.22516(4)	0.50189(4)	0.00963(6)
Cl(1)	4*a*	0.56853(3)	0.77234(17)	0.46699(11)	0.01819(18)
Cl(2)	4*a*	0.31949(3)	0.03073(18)	0.45880(11)	0.01803(17)
O/F(1A)	4*a*	0.56433(8)	0.8717(5)	0.6348(3)	0.0198(6)
O/F(1B)	4*a*	0.54739(7)	0.5131(4)	0.4807(3)	0.0162(6)
O/F(1C)	4*a*	0.53404(8)	0.9228(5)	0.3789(3)	0.0190(6)
O(2)	4*a*	0.29393(8)	−0.2095(4)	0.4738(3)	0.0119(5)
F(2)	4*a*	0.28851(8)	0.2040(5)	0.3668(3)	0.0218(5)
F(3)	4*a*	0.31588(8)	0.1416(5)	0.6254(3)	0.0199(4)
F(4)	4*a*	0.38612(7)	0.8110(4)	0.5525(3)	0.0175(4)
F(5)	4*a*	0.40645(7)	0.3793(4)	0.7077(2)	0.0169(4)
F(6)	4*a*	0.37546(8)	0.3686(4)	0.4128(3)	0.0180(4)
F(7)	4*a*	0.47015(7)	0.6989(5)	0.6234(3)	0.0188(4)
F(8)	4*a*	0.44239(8)	0.6738(4)	0.3156(3)	0.0210(5)
F(9)	4*a*	0.46102(7)	0.2516(4)	0.4717(3)	0.0223(6)
F(10)	4*a*	0.22156(7)	0.0760(4)	0.6077(3)	0.0191(4)
F(11)	4*a*	0.13808(7)	−0.0650(4)	0.5504(3)	0.0159(4)
F(12)	4*a*	0.15597(7)	0.3711(5)	0.7032(3)	0.0178(4)
F(13)	4*a*	0.18939(8)	0.0912(4)	0.3038(3)	0.0194(4)
F(14)	4*a*	0.20623(8)	0.5167(4)	0.4587(3)	0.0219(5)
F(15)	4*a*	0.12105(7)	0.3745(4)	0.4155(3)	0.0164(4)

Table S14. Anisotropic displacement parameters of $ClOF_2[NbF_6]$ at 100 K.

Atom	U_{11} / Å^2	U_{22} / Å^2	U_{33} / Å^2	U_{23} / Å^2	U_{13} / Å^2	U_{12} / Å^2
Nb(1)	0.00898(11)	0.01095(10)	0.00972(12)	0.00031(14)	0.00020(13)	−0.00001(8)
Nb(2)	0.00904(10)	0.01078(10)	0.00907(11)	−0.00162(14)	−0.00011(13)	−0.00003(8)
Cl(1)	0.0109(3)	0.0223(4)	0.0214(5)	−0.0017(3)	0.0047(3)	−0.0007(3)
Cl(2)	0.0108(3)	0.0254(4)	0.0179(4)	−0.0025(3)	0.0025(3)	0.0006(3)
O/F(1A)	0.0230(13)	0.0233(12)	0.0131(11)	−0.0053(9)	0.0041(9)	−0.0003(10)
O/F(1B)	0.0148(10)	0.0129(9)	0.0208(14)	−0.0003(8)	0.0034(8)	−0.0035(7)
O/F(1C)	0.0140(12)	0.0194(11)	0.0235(13)	0.0030(9)	−0.0025(9)	0.0054(9)
O(2)	0.0127(9)	0.0098(8)	0.0131(13)	−0.0008(8)	0.0035(8)	−0.0049(8)
F(2)	0.0167(11)	0.0235(11)	0.0252(12)	0.0058(9)	−0.0024(9)	0.0049(9)
F(3)	0.0195(11)	0.0248(11)	0.0153(10)	−0.0049(9)	0.0032(9)	0.0005(9)
F(4)	0.0154(10)	0.0166(9)	0.0204(11)	−0.0002(8)	−0.0013(8)	0.0058(8)
F(5)	0.0170(10)	0.0226(11)	0.0110(10)	0.0058(8)	−0.0004(8)	−0.0007(8)
F(6)	0.0188(11)	0.0205(10)	0.0148(10)	0.0018(8)	−0.0052(8)	−0.0079(8)
F(7)	0.0118(10)	0.0222(10)	0.0224(11)	−0.0062(9)	−0.0029(8)	−0.0018(8)
F(8)	0.0249(12)	0.0218(11)	0.0163(11)	0.0045(9)	0.0058(9)	−0.0039(9)
F(9)	0.0199(10)	0.0164(10)	0.0305(16)	−0.0055(8)	0.0028(10)	0.0052(8)
F(10)	0.0116(10)	0.0244(11)	0.0213(11)	0.0038(9)	−0.0039(8)	0.0028(8)
F(11)	0.0148(9)	0.0139(9)	0.0190(11)	0.0002(7)	−0.0003(7)	−0.0033(7)
F(12)	0.0179(10)	0.0238(11)	0.0117(10)	−0.0071(8)	0.0002(8)	0.0008(9)
F(13)	0.0219(11)	0.0230(11)	0.0134(10)	−0.0056(8)	0.0028(9)	0.0006(9)
F(14)	0.0228(11)	0.0156(10)	0.0272(13)	0.0003(8)	0.0038(9)	−0.0052(8)
F(15)	0.0169(10)	0.0171(10)	0.0152(10)	−0.0025(8)	−0.0049(8)	0.0054(8)

Atomic coordinates and displacement parameters of $ClOF_2[TaF_6]$

Table S15. Atomic coordinates and equivalent isotropic displacement parameters U_{iso} of $ClOF_2[TaF_6]$ at 100 K.

Atom	Position	*x*	*y*	*z*	U_{iso} / Å^2
Ta(1)	4*a*	0.42478(2)	0.53075(3)	0.51004(3)	0.01523(5)
Ta(2)	4*a*	0.17310(2)	0.22083(3)	0.50208(3)	0.01507(5)
Cl(1)	4*a*	0.56802(5)	0.7773(3)	0.4680(2)	0.0248(3)
Cl(2)	4*a*	0.31896(5)	0.0287(3)	0.4591(2)	0.0243(3)
O/F(1A)	4*a*	0.56345(15)	0.8735(8)	0.6344(5)	0.0233(9)
O/F(1B)	4*a*	0.53376(15)	0.9237(8)	0.3778(5)	0.0269(10)
O/F(3A)	4*a*	0.29342(13)	−0.2117(8)	0.4730(5)	0.0270(11)
O/F(3B)	4*a*	0.28817(14)	0.1994(8)	0.3670(5)	0.0244(9)
O/F(3C)	4*a*	0.31529(14)	0.1380(8)	0.6257(5)	0.0235(9)
F(2)	4*a*	0.54680(12)	0.5174(7)	0.4794(5)	0.0287(9)
F(4)	4*a*	0.38572(12)	0.8065(6)	0.5519(4)	0.0221(7)
F(5)	4*a*	0.40669(13)	0.3793(7)	0.7081(4)	0.0238(7)
F(6)	4*a*	0.37506(13)	0.3638(7)	0.4141(5)	0.0249(8)
F(7)	4*a*	0.46994(13)	0.6978(8)	0.6208(5)	0.0260(8)
F(8)	4*a*	0.44145(15)	0.6698(8)	0.3135(5)	0.0291(8)
F(9)	4*a*	0.46066(14)	0.2492(6)	0.4720(5)	0.0312(10)
F(10)	4*a*	0.22115(12)	0.0704(7)	0.6046(5)	0.0250(8)
F(11)	4*a*	0.13729(12)	−0.0684(6)	0.5506(4)	0.0209(7)
F(12)	4*a*	0.15608(13)	0.3655(7)	0.7038(4)	0.0246(8)
F(13)	4*a*	0.18835(14)	0.0891(7)	0.3020(5)	0.0267(8)
F(14)	4*a*	0.20568(14)	0.5115(7)	0.4583(5)	0.0280(8)
F(15)	4*a*	0.12052(13)	0.3712(7)	0.4162(5)	0.0235(7)

Table S16. Anisotropic displacement parameters of $ClOF_2[TaF_6]$ at 100 K.

Atom	U_{11} / Å^2	U_{22} / Å^2	U_{33} / Å^2	U_{23} / Å^2	U_{13} / Å^2	U_{12} / Å^2
Ta(1)	0.01457(9)	0.01628(8)	0.01484(9)	0.00053(12)	0.00061(13)	0.00048(5)
Ta(2)	0.01484(9)	0.01647(8)	0.01389(9)	−0.00175(12)	0.00019(13)	0.00049(6)
Cl(1)	0.0182(6)	0.0274(6)	0.0288(9)	−0.0026(5)	0.0052(5)	−0.0005(5)
Cl(2)	0.0165(6)	0.0316(7)	0.0247(7)	−0.0031(5)	0.0031(5)	0.0013(5)
O/F(1A)	0.028(2)	0.026(2)	0.0156(18)	−0.0049(16)	0.0033(16)	0.0009(17)
O/F(1B)	0.025(2)	0.026(2)	0.029(2)	0.0031(17)	−0.0032(18)	0.0062(16)
O/F(3A)	0.0268(18)	0.0233(16)	0.031(3)	0.0002(16)	0.0030(17)	−0.0037(14)
O/F(3B)	0.0191(19)	0.029(2)	0.025(2)	0.0062(16)	−0.0026(16)	0.0052(16)
O/F(3C)	0.027(2)	0.026(2)	0.0176(18)	−0.0051(15)	0.0018(16)	0.0017(16)
F(2)	0.0259(17)	0.0244(16)	0.036(3)	0.0009(17)	0.0057(17)	−0.0041(12)
F(4)	0.0226(17)	0.0206(15)	0.0230(18)	0.0004(13)	−0.0008(13)	0.0057(13)
F(5)	0.0244(18)	0.0306(19)	0.0164(16)	0.0069(14)	0.0005(14)	0.0019(15)
F(6)	0.0270(19)	0.0247(18)	0.0228(18)	0.0001(15)	−0.0056(15)	−0.0062(14)
F(7)	0.0185(18)	0.0296(18)	0.030(2)	−0.0048(16)	−0.0021(15)	−0.0016(15)
F(8)	0.035(2)	0.030(2)	0.0219(18)	0.0063(16)	0.0081(17)	−0.0017(17)
F(9)	0.035(2)	0.0212(18)	0.038(3)	−0.0030(15)	0.010(2)	0.0104(13)
F(10)	0.0173(17)	0.0293(19)	0.029(2)	0.0034(16)	−0.0039(15)	0.0031(14)
F(11)	0.0207(16)	0.0182(14)	0.0238(18)	0.0007(12)	−0.0022(13)	−0.0035(12)
F(12)	0.0270(19)	0.0297(19)	0.0172(17)	−0.0093(14)	0.0005(15)	−0.0010(15)
F(13)	0.034(2)	0.0284(19)	0.0177(17)	−0.0077(15)	0.0048(16)	0.0021(16)
F(14)	0.0297(19)	0.0225(17)	0.032(2)	0.0012(14)	0.0040(17)	−0.0081(14)
F(15)	0.0252(19)	0.0216(17)	0.0237(17)	−0.0013(14)	−0.0054(14)	0.0075(14)

Plot of distances and angles within the $ClOF_2^+$ cations versus effective ionic radii of *M*(V) ions

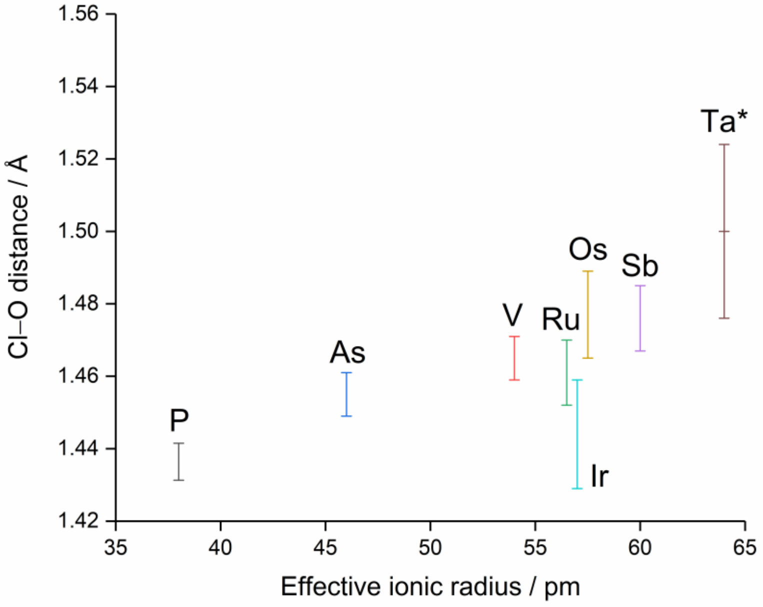

Figure S1. Plot of experimentally determined Cl−O distances obtained from single-crystal X-ray diffraction of the $ClOF_2^+$ cations versus effective ionic radii of *M*(V) ions.[1] The values of $ClOF_2[AsF_6]$ were taken from a previous study.[2] For $ClOF_2[TaF_6]$, the minimal and maximal Cl−O/F distances of the disordered cations were taken. Distances of $ClOF_2[NbF_6]$ are not plotted, as the values are very close to those of $ClOF_2[TaF_6]$. The error bars indicate the tripled standard uncertainties.

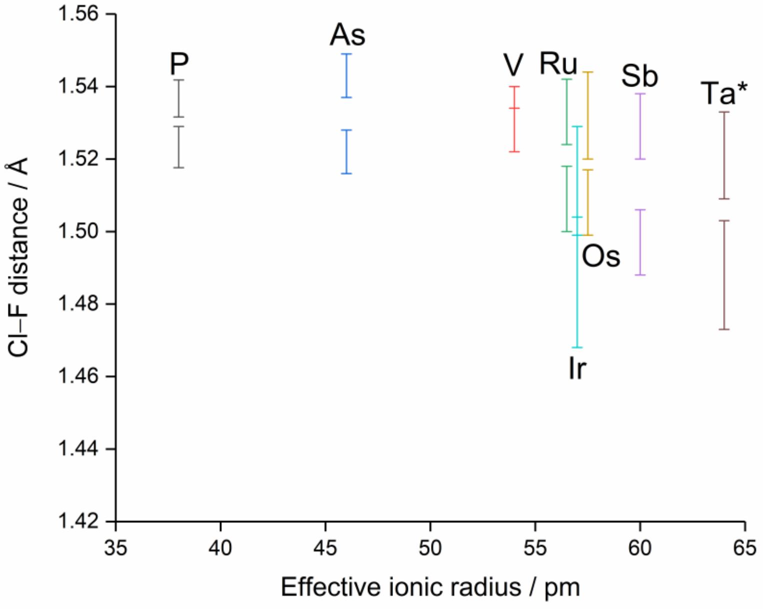

Figure S2. Plot of experimentally determined Cl−F distances obtained from single-crystal X-ray diffraction of the $ClOF_2^+$ cations versus effective ionic radii of *M*(V) ions.[1] The values of $ClOF_2[AsF_6]$ were taken from a previous study.[2] For $ClOF_2[TaF_6]$, the minimal and maximal Cl−O/F and Cl−F distances of the fully/partially disordered cations were taken. Distances of $ClOF_2[NbF_6]$ are not plotted, as the values are very close to those of $ClOF_2[TaF_6]$. The error bars indicate the tripled standard uncertainties.

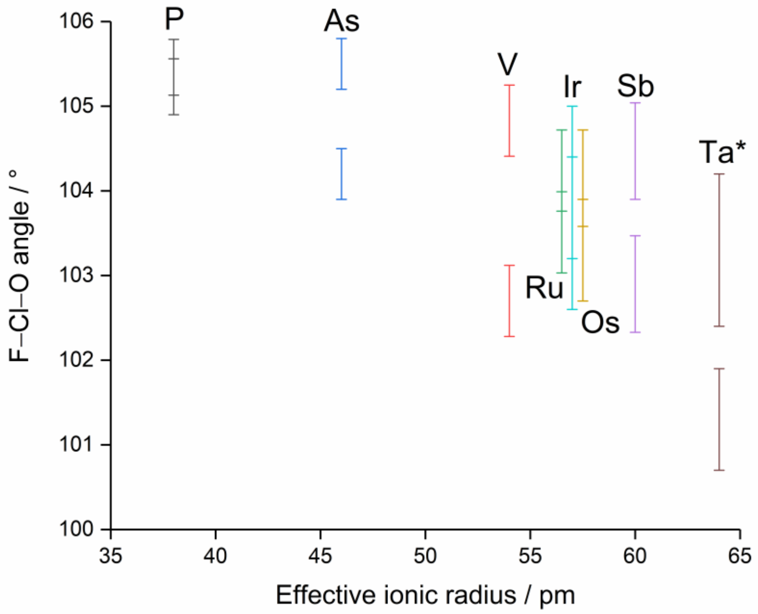

Figure S3. Plot of experimentally determined F−Cl−O angles obtained from single-crystal X-ray diffraction of the $ClOF_2^+$ cations versus effective ionic radii of *M*(V) ions.[1] The values of $ClOF_2[AsF_6]$ were taken from a previous study.[2] For $ClOF_2[TaF_6]$, the minimal and maximal O/F−Cl−O/F and O/F−Cl−F angles of the fully/partially disordered cations were taken. Angles of $ClOF_2[NbF_6]$ are not plotted, as the values are very close to those of $ClOF_2[TaF_6]$. The error bars indicate the tripled standard uncertainties.

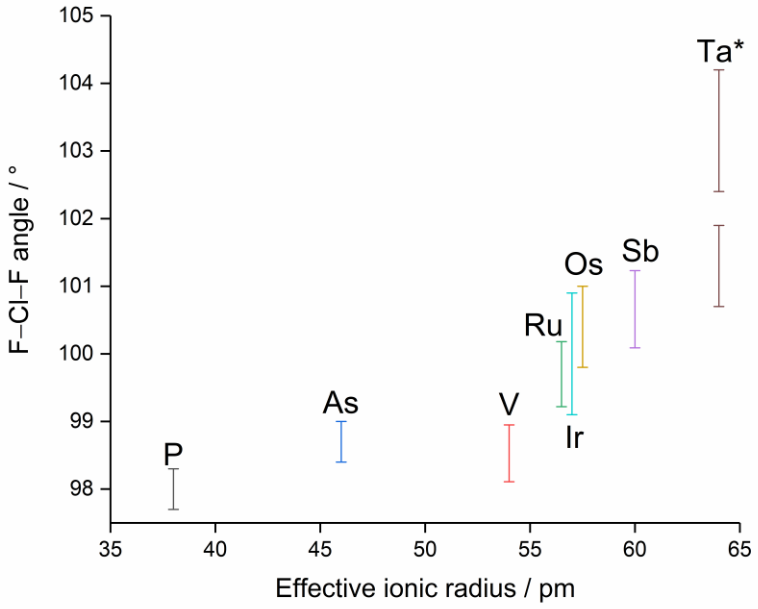

Figure S4. Plot of experimentally determined F−Cl−F angles obtained from single-crystal X-ray diffraction of the $ClOF_2^+$ cations versus effective ionic radii of *M*(V) ions.[1] The values of $ClOF_2[AsF_6]$ were taken from a previous study.[2] For $ClOF_2[TaF_6]$, the minimal and maximal O/F−Cl−O/F and O/F−Cl−F angles of the fully/partially disordered cations were taken. Angles of $ClOF_2[NbF_6]$ are not plotted, as the values are very close to those of $ClOF_2[TaF_6]$. The error bars indicate the tripled standard uncertainties.

Basis set details for quantum-chemical solid-state calculations

TZVP basis set for O

The basis set for O was taken from a previous study.[3]

TZVP basis set for F

The basis set for F was taken from a previous study.[3]

TZVP basis set for Cl

The basis set for Cl was taken from a previous study.[4]

TZVP basis set for V

The basis set for V was taken from a previous study.[5]

TZVP basis set for As

The starting point was the molecular def-TZVP basis set.[6] The exponents of the outermost s and p functions were fixed to 0.13 and the exponents of the other s and p functions in the valence space were reoptimized for the arsenic atom in its ground state. The exponent of the outermost d primitive was kept fixed, while the exponents of the other d functions were optimised for the arsenic atom in its ground state. An f-type polarization function with an exponent of 0.433 was added (def2-TZVP basis set). Finally, the outermost s and p functions were combined into a single sp-type function to increase the efficiency of the CRYSTAL code. The resulting energy loss w.r.t unmodified def2-TZVP basis set is 0.2 mH. The final basis set in CRYSTAL input format is as follows:

```
33 14
0 0 8 2.0 1.0
  498032.42158       0.22740196900E-03
  74656.868743       0.17632816413E-02
  16990.960004       0.91728040381E-02
  4809.6200321       0.37337829344E-01
  1566.2887055       0.12199536117
  563.21360499       0.29137475324
  219.11179978       0.42326351479
  86.866061030       0.22921464278
0 0 4 2.0 1.0
  538.19512479      -0.25254197297E-01
  167.14850224      -0.11915461115
  27.605517159       0.54628495980
  11.947858521       0.53001520976
0 0 2 2.0 1.0
  18.538023133      -0.23479188136
  3.2018985739       0.69167053428
0 0 1 2.0 1.0
  1.4362581800        1.0000000000
0 0 1 0.0 1.0
 0.35310762320        1.0000000000
0 1 1 0.0 1.0
 0.13000000000        1.0 1.0
0 2 6 6.0 1.0
  2678.9421546       0.23318955287E-02
  634.61765840       0.19042149977E-01
  203.93967606       0.90229744913E-01
  76.323890369       0.26169037693
  30.664124943       0.41857168155
  12.505056732       0.23447830190
0 2 4 6.0 1.0
  49.256229549      -0.21235539870E-01
  7.7274891466       0.30470206668
  3.5410493476       0.52888373107
  1.6985585501       0.37272250955
0 2 1 3.0 1.0
 0.80396418504        1.0000000000
0 2 1 0.0 1.0
 0.41045871535        1.0000000000
0 3 5 10.0 1.0
  139.05230680       0.99291144106E-02
  41.113850141       0.66568843496E-01
  15.035409461       0.22275768307
  5.9891096071       0.40309224382
  2.4322147899       0.41671667946
0 3 1 0.0 1.0
 0.96247584610        1.0000000000
0 3 1 0.0 1.0
 0.29300000000        1.0000000000
0 4 1 0.0 1.0
```

```
 0.43271941          1.0
```

TZVP basis set for Sb

The def2-TZVP basis set with a 28-electron effective core potential was used as a starting point.[6] We fixed the exponents of the outermost s and p functions to 0.11 and reoptimized the exponents of the other s and p functions in the valence space for the antimony atom in its ground state. Finally, the outermost s and p functions were combined into one sp-type function. The resulting energy loss with respect to the original molecular basis set is 4.7 mH. The exponent of the outermost d-function was increased from 0.14 to 0.217. The energy cost of this change was only 0.14 mH. The steep f-type polarization function with an exponent of 1.1 was removed. The final basis set in CRYSTAL input format is as follows:

```
251 13
INPUT
23. 0 2 4 4 2 0
16.330865  281.071581 0
8.556542    61.716604 0
14.470337   67.457380 0
13.816194  134.933503 0
8.424924    14.716344 0
8.092728    29.518512 0
14.886331   35.447815 0
15.146319   53.143466 0
5.908267     9.179223 0
5.594322    13.240253 0
14.444978  -15.366801 0
14.449295  -20.296138 0
0 0 4 2.0 1.0
  1612.4199933      0.28540380783E-03
  238.84452097      0.13393778746E-02
  23.998118809     -0.49388154574E-01
  15.193124213      0.43392227254
0 0 2 2.0 1.0
  11.731361334      0.92125519965
  6.5268387226      0.79235280226
0 0 1 0.0 1.0
 2.0178590021       1.0000000000
0 0 1 0.0 1.0
 0.96219277446       1.0000000000
0 0 1 0.0 1.0
 0.28581551962       1.0000000000
0 1 1 0.0 1.0
 0.11                1.0 1.0
0 2 3 6.0 1.0
  215.68393354      0.26051823221E-03
  16.374479088      0.73728000195E-01
  9.7216283345     -0.27230028128
0 2 3 3.0 1.0
  2.7979771259      0.46472692374
  1.4696586758      0.50364242075
 0.74906751891      0.18706666294
0 2 1 0.0 1.0
  0.32300924430     1.0000000000
0 3 6 10.0 1.0
  115.90312253      0.53140915051E-03
  30.474233720      0.59411139166E-02
  18.228418239     -0.10563706947E-01
  4.3291456646      0.20348177341
  2.1294818496      0.42748378928
 0.99682636692      0.38539560809
0 3 1 0.0 1.0
 0.43347239863      1.0000000000
0 3 1 0.0 1.0
 0.216736199315     1.0000000000
0 4 1 0.0 1.0
 0.32369706         1.0000000000
```

TZVP basis set for Nb

The basis set was derived from the molecular Karlsruhe def2-TZVP basis set (28-electron effective core potential).[6] The diffuse outermost s-exponents were increased from 0.033 and 0.086 to 0.13 and 0.30, respectively. The outermost p-type function with an exponent of 0.03 was removed and the exponents of the two remaining outermost p-type functions were changed from 0.09 to 0.13 and 0.2797 to 0.30. The two outermost s and p functions were both then combined into two sp-type functions. The most diffuse d-type function (exponent 0.10) was removed and the inner (3d) function was decontracted to (2d1d). The f-type polarization function was removed. The final basis set in CRYSTAL input format is as follows:

```
241 9
INPUT
13. 0 2 2 2 2 0
8.900000 165.179143 0
4.430000 21.992974 0
7.770000 111.794414 0
3.960000 16.633483 0
6.050000 38.112249 0
2.840000 8.039167 0
8.490000 -22.929550 0
4.250000 -3.666310 0
0 0 2 2.0 1.0
  12.000000000     -0.20219507530
  10.500000000      0.33640105939
0 0 1 2.0 1.0
  3.9276062854      1.0000000000
0 0 1 0.0 1.0
 0.85976543258      1.0000000000
0 1 1 0.0 1.0
 0.30               1.0 1.0
0 1 1 0.0 1.0
 0.13               1.0 1.0
0 2 4 6.0 1.0
  9.2056285646      0.43347689874E-01
  4.8679632125     -0.21302479233
  1.2442155792      0.48102127136
 0.60390590312      0.53917858960
0 3 2 3.0 1.0
  4.6170975867     -0.13574476955E-01
  1.5663438480      0.20374310496
0 3 1 0.0 1.0
 0.66952425826      0.42997453105
0 3 1 0.0 1.0
 0.27140946867      1.0000000000
```

TZVP basis set for Ta

The basis set was derived from the molecular Karlsruhe def2-TZVP basis set (60-electron effective core potential).[6] The diffuse outermost s- and p-type functions with exponents of 0.039 and 0.065 were removed. The exponent of the outermost s-type function was increased from 0.10 and 0.11. The exponent of the outermost p-type function was changed from 0.27 to 0.11 and combined with the outermost s function into a single sp-type function. The most diffuse d-type function (exponent 0.088) was removed and the inner (3d) function was decontracted to (2d1d). The f-type polarization function was removed. The final basis set in CRYSTAL input format is as follows:

```
273 10
INPUT
13. 0 2 2 2 1 1
14.546408 1345.880647 0
7.273204 36.766806 0
9.935565 378.425301 0
4.967782 22.293091 0
6.347377 104.883956 0
3.173688 8.755848 0
2.017881 12.017961 0
3.040330 -11.728933 0
0 0 4 2.0 1.0
  24.473650944      0.20590322488
  18.721372549     -0.74670795514
  11.500000000       3.4071363897
  10.350000000      -2.8175487609
0 0 1 2.0 1.0
  3.8436180089       1.0000000000
0 0 1 0.0 1.0
  1.0202266016       1.0000000000
0 0 1 0.0 1.0
 0.46774781869       1.0000000000
0 1 1 0.0 1.0
  0.11              1.0 1.0
0 2 4 6.0 1.0
  17.000000000     -0.32577305616E-01
  12.008186536      0.10336287365
  5.0278760583     -0.28526521696
  1.1937124184      0.51790141155
0 2 1 0.0 1.0
 0.57889707053       1.0000000000
```

```
0 3 2 3.0 1.0
  3.9738796278     -0.52799310714E-01
  1.4528884813      0.18558319471
0 3 1 0.0 1.0
 0.61042908544      0.42959071631
0 3 1 0.0 1.0
 0.24216276510       1.0000000000
```

TZVP basis set for Ir

The basis set was derived from the molecular Karlsruhe def2-TZVP basis set (60-electron effective core potential).[6] The diffuse outermost s- and p-type functions with exponents of 0.05 and 0.056 were removed. The exponent of the outermost p-type function was changed from 0.33 to 0.14 and it was combined with the outermost s function into a single sp-type function. The exponents of the two outermost d-type functions were increased to 0.18 and 0.36. The f-type polarization function was removed. The final basis set in CRYSTAL input format is as follows:

```
277 10
INPUT
17. 0 2 2 2 1 1
13.652203 732.269200 0
6.826101 26.484721 0
10.279868 299.489474 0
5.139934 26.466234 0
7.349859 124.457595 0
3.674929 14.035995 0
3.034072 21.531031 0
4.808857 -21.607597 0
0 0 3 2.0 1.0
  30.000000000      0.30797903228
  27.000000000     -0.46726361781
  13.961973911      0.47161003146
0 0 1 2.0 1.0
  5.3956977802       1.0000000000
0 0 1 0.0 1.0
  1.2149128721       1.0000000000
0 0 1 0.0 1.0
 0.55885743756       1.0000000000
0 1 1 0.0 1.0
 0.14097974313       1.0 1.0
0 2 4 6.0 1.0
  15.902664143     -0.16290720099
  14.415830698      0.23483212987
  5.7597608991     -0.30305337176
  1.5008913108      0.55512982069
0 2 1 0.0 1.0
 0.72348035957       1.0000000000
0 3 4 7.0 1.0
  8.6321692504      0.75000099949E-01
  6.5898192302     -0.17326965173
  1.5808379663      0.55065196913
 0.71827834905      0.85273641436
0 3 1 0.0 1.0
 0.36                1.0000000000
0 3 1 0.0 1.0
 0.18                1.0000000000
```

TZVP basis set for Ru

The basis set was derived from the molecular Karlsruhe def2-TZVP basis set (28-electron effective core potential).[6] The diffuse outermost s-exponents were increased from 0.039 and 0.107 to 0.13 and 0.26, respectively. The outermost p-type function with an exponent of 0.037 was removed and the exponents of the two remaining outermost p-type functions were changed from 0.115 to 0.13 and 0.367 to 0.26. The two outermost s and p functions were then combined into two sp-type functions. The exponent of the outermost d-type function (exponent 0.15) increased to 0.20. The f-type polarization function was removed. The final basis set in CRYSTAL input format is as follows:

```
244 10
INPUT
16. 0 2 2 2 2 0
11.105269 209.822971 0
5.414745 30.654726 0
9.771271 146.336182 0
5.073991 24.127877 0
7.671423 67.515897 0
4.136565 9.870104 0
11.360000 -28.340616 0
5.680000 -4.944629 0
0 0 2 2.0 1.0
  16.000000000     -0.20634002919
  13.910581694      0.33550437725
0 0 1 2.0 1.0
  4.7967971407       1.0000000000
0 0 1 0.0 1.0
  1.1555425594       1.0000000000
0 0 1 0.0 1.0
```

```
 0.52455741182       1.0000000000
0 1 1 0.0 1.0
 0.26               1.0 1.0
0 1 1 0.0 1.0
 0.13               1.0 1.0
0 2 4 6.0 1.0
  11.187208671      0.53225073263E-01
  6.2477688734     -0.22731662050
  1.6279472859      0.47869486056
 0.79326493538      0.50213311567
0 3 3 6.0 1.0
  5.7341846619     -0.35266111560E-01
  2.2483686294      0.21802502222
 0.98376978359      0.44709565013
0 3 1 0.0 1.0
 0.40379445583      1.0000000000
0 3 1 0.0 1.0
 0.2                1.0000000000
```

TZVP basis set for Os

The basis set was derived from the molecular Karlsruhe def2-TZVP basis set (60-electron effective core potential).[6] The diffuse outermost s- and p-type functions with exponents of 0.047 and 0.052 were removed. The exponent of the outermost p-type function was changed from 0.31 to 0.13 and it was combined with the outermost s function into a single sp-type function. The most diffuse d-type function (exponent 0.11) was removed and the inner (4d) function was decontracted to (3d1d). The f-type polarization function was removed. The final basis set in CRYSTAL input format is as follows:

```
276 10
INPUT
16. 0 2 2 2 1 1
13.875754 885.405719 0
6.937877 25.967040 0
10.193793 320.083902 0
5.096896 26.148765 0
7.099238 115.044843 0
3.549619 13.622575 0
2.767075 18.909457 0
4.349905 -19.027595 0
0 0 3 2.0 1.0
  30.000000000      0.32213004804
  27.000000000     -0.47922918137
  13.524730005      0.47465946837
0 0 1 2.0 1.0
  5.2402883472       1.0000000000
0 0 1 0.0 1.0
  1.1306661245       1.0000000000
0 0 1 0.0 1.0
 0.51689966917       1.0000000000
0 1 1 0.0 1.0
 0.13222953545       1.0 1.0
0 2 4 6.0 1.0
  15.500000000      0.16016463376
  14.000000000     -0.23201520402
  5.5458290420      0.29772587274
  1.4139502215     -0.54253238176
0 2 1 0.0 1.0
 0.68136686765       1.0000000000
0 3 3 6.0 1.0
  8.2945059487      0.69648222110E-01
  6.3060397430     -0.16444229630
  1.4890109110      0.55154541033
0 3 1 0.0 1.0
 0.67315390607      0.88461370291
0 3 1 0.0 1.0
 0.22               1.0000000000
```

Lattice parameters and atomic coordinates of optimized solid-state structures of $ClOF_2[MF_6]$ (*M* = V, Nb, Ta, Ru, Os, Ir, P, As, Sb)

Lattice parameters and atomic coordinates of the optimized solid-state structure of $ClOF_2[PF_6]$

Space group $Pna2_1$ (No. 33)
a = 14.38025897 Å, b = 5.08846086 Å, c = 8.04744210 Å, V = 588.858579 Å^3

Table S17. Atomic coordinates of the optimized structure of $ClOF_2[PF_6]$.

Atom	*x*	*y*	*z*
P	4.062778554519E−01	3.864604967303E−01	−2.709139186074E−01
Cl	3.868244093407E−01	1.431994641732E−01	2.804371365093E−01
O	3.319807886150E−01	3.713200620399E−01	2.641350018282E−01
F	3.242535740893E−01	−7.163199477015E−02	3.785570542197E−01
F	3.781576290736E−01	−1.178668638641E−02	1.056809994518E−01
F	3.474471715323E−01	1.261369234349E−01	−2.291718044716E−01
F	3.189907454284E−01	−4.870781340849E−01	−3.695082726865E−01
F	4.419116123237E−01	2.484687947995E−01	−4.477141076297E−01
F	4.693615542443E−01	−3.537791654662E−01	−3.200683413392E−01
F	4.985362320363E−01	2.603065159246E−01	−1.808395844815E−01
F	3.755698206090E−01	−4.766600226452E−01	−9.933416279322E−02

Lattice parameters and atomic coordinates of the optimized solid-state structure of $ClOF_2[VF_6]$

Space group $Pna2_1$ (No. 33)
a = 14.83986594 Å, b = 5.12465376 Å, c = 8.27596028 Å, V = 629.379950 Å^3

Table S18. Atomic coordinates of the optimized structure of $ClOF_2[VF_6]$.

Atom	*x*	*y*	*z*
V	4.024998290335E−01	3.937739568582E−01	−2.801753905208E−01
Cl	3.924995245221E−01	1.478612410209E−01	2.706943344949E−01
O	3.424253932990E−01	3.805299848763E−01	2.537131057854E−01
F	3.249706786827E−01	−5.890449197671E−02	3.598530406277E−01
F	3.819653201076E−01	−1.220876943674E−03	9.786716679915E−02
F	3.135760380775E−01	−4.593801701868E−01	−3.854384080866E−01
F	3.394864720533E−01	1.160129713872E−01	−2.390124736320E−01
F	4.742453974179E−01	−3.285355864486E−01	−3.332821997414E−01
F	4.384012046849E−01	2.449971621154E−01	−4.735618915157E−01
F	3.737701582601E−01	−4.601780842940E−01	−9.820659039276E−02
F	−4.990168701804E−01	2.481531848385E−01	−1.935606938180E−01

Lattice parameters and atomic coordinates of the optimized solid-state structure of $ClOF_2[AsF_6]$

The initial parameters for the optimization were taken from a previous study on the crystal structure of $ClOF_2[AsF_6]$.[2]

Space group *Pna*2_1 (No. 33)
a = 14.63689586 Å, *b* = 5.10684533 Å, *c* = 8.16955416 Å, *V* = 610.660802 $Å^3$

Table S19. Atomic coordinates of the optimized structure of $ClOF_2[AsF_6]$.

Atom	*x*	*y*	*z*
As	9.361603309681E−02	3.877779212081E−01	1.407934084638E−01
Cl	1.137674315014E−01	1.443557424744E−01	−4.086145687992E−01
O	1.659085132510E−01	3.750982333500E−01	−3.926861526771E−01
F	−3.300954769340E−03	2.531855068144E−01	5.001142866646E−02
F	2.707357999303E−02	−3.390681248614E−01	1.925799618570E−01
F	5.755557699395E−02	2.436489846825E−01	3.264741000832E−01
F	1.550559099730E−01	1.119971681585E−01	9.752801597998E−02
F	1.843951808439E−01	−4.760404152474E−01	2.434188484937E−01
F	3.220920134006E−01	4.340715173744E−01	−3.933188277927E−03
F	3.757323073108E−01	3.129579804577E−02	4.599798774131E−01
F	3.770448115688E−01	4.915777576132E−01	2.642782687969E−01

Lattice parameters and atomic coordinates of the optimized solid-state structures of $ClOF_2[NbF_6]$

Model 1 (full oxygen occupation on position O(1A))

Energy difference relative to Model 3: 6.6 kJ/mol

Space group *Pna*2_1 (No. 33)
a = 30.32836377 Å, *b* = 5.23609528 Å, *c* = 8.45914808 Å, *V* = 1343.331347 $Å^3$

Table S20. Atomic coordinates of the optimized structure of $ClOF_2[NbF_6]$. (full oxygen occupation on position O(1A))

Atom	*x*	*y*	*z*
Nb	4.232274639452E−01	−4.741423061017E−01	−4.847162508938E−01
Nb	1.717776174416E−01	2.378287439949E−01	4.959365967078E−01
Cl	−4.310239782525E−01	−2.317179064533E−01	4.842073428372E−01
Cl	3.195935706686E−01	−7.683329970997E−03	4.541376706808E−01
O	−4.379782135432E−01	−1.436164773254E−01	−3.602351218474E−01
O	2.947084671817E−01	−2.348086997231E−01	4.662033179675E−01
F	−4.682685251491E−01	−9.191470030614E−02	3.758781615374E−01
F	2.867330095788E−01	1.985486904815E−01	3.675037526893E−01
F	−4.559905605881E−01	4.920113435762E−01	4.726051986008E−01
F	3.143649461809E−01	1.330448786616E−01	−3.754911927144E−01
F	3.834670480343E−01	−1.837928430226E−01	−4.452417297253E−01
F	4.060110271317E−01	3.876264871981E−01	−2.818461083765E−01
F	3.718643391636E−01	3.625234553947E−01	4.247942265021E−01
F	4.379166233748E−01	−3.421091652663E−01	3.144689773123E−01
F	4.694344737392E−01	−2.967840823168E−01	−3.835569498425E−01
F	2.201148245075E−01	8.247903889347E−02	−4.035230730011E−01
F	1.344085729118E−01	−5.125135884368E−02	−4.491218522782E−01
F	1.567076941377E−01	3.881686040143E−01	−2.983784415613E−01
F	1.852082376857E−01	1.045732394568E−01	2.962544767186E−01
F	2.027446107961E−01	−4.605658321338E−01	4.509004048581E−01
F	1.171030546968E−01	3.925485376831E−01	4.212132763261E−01
F	4.577910074127E−01	2.343334363421E−01	4.792973175025E−01

Lattice parameters and atomic coordinates of the optimized solid-state structures of $ClOF_2[NbF_6]$

Model 2 (full oxygen occupation on position O(1B))

Energy difference relative to Model 3: 6.0 kJ/mol

Space group *Pna*2$_1$ (No. 33)
a = 30.60513126 Å, *b* = 5.17841765 Å, *c* = 8.50529152 Å, *V* = 1347.970923 Å^3

Table S21. Atomic coordinates of the optimized structure of $ClOF_2[NbF_6]$. (full oxygen occupation on position O(1B))

Atom	*x*	*y*	*z*
Nb	4.229791707176E−01	−4.724010081077E−01	−4.952435232552E−01
Nb	1.726714892951E−01	2.412234140905E−01	−4.965366792128E−01
Cl	−4.314683452136E−01	−2.823565727950E−01	4.608922778962E−01
Cl	3.187258057045E−01	−8.741611494593E−03	4.606243867571E−01
O	2.946577283381E−01	−2.398254326739E−01	4.761840087935E−01
O	−4.534798343798E−01	4.787178461563E−01	4.730257608633E−01
F	−4.372813251818E−01	−1.440055126230E−01	−3.685070310017E−01
F	−4.662282346185E−01	−8.563270053139E−02	3.781648328090E−01
F	2.858239442573E−01	1.961583746758E−01	3.739386096608E−01
F	3.139989921629E−01	1.375105992617E−01	−3.708431134552E−01
F	3.844057911691E−01	−1.829532212109E−01	−4.436923230643E−01
F	4.079170170285E−01	3.766313526967E−01	−2.900472797954E−01
F	3.708028899258E−01	3.669705722341E−01	4.239395488084E−01
F	4.369483753179E−01	−3.295054433111E−01	3.084552128180E−01
F	4.697367327872E−01	−3.074200198248E−01	−3.912092685597E−01
F	2.203392737251E−01	7.731451369798E−02	−3.975041494055E−01
F	1.348954923362E−01	−5.193544249165E−02	−4.447534200711E−01
F	1.583434654047E−01	3.857475909264E−01	−2.890409239568E−01
F	1.854670546271E−01	1.029899853426E−01	3.048689578273E−01
F	2.047868665520E−01	−4.588557951756E−01	4.595884406436E−01
F	1.198097310822E−01	4.008746262185E−01	4.277310993401E−01
F	4.557119154256E−01	2.297277352011E−01	4.612545755605E−01

Lattice parameters and atomic coordinates of the optimized solid-state structures of $ClOF_2[NbF_6]$

Model 3 (full oxygen occupation on position O(1C))

Space group $Pna2_1$ (No. 33)
a = 30.58152116 Å, b = 5.22196635 Å, c = 8.46186047 Å, V = 1351.322514 Å^3

Table S22. Atomic coordinates of the optimized structure of $ClOF_2[NbF_6]$. (full oxygen occupation on position O(1C))

Atom	*x*	*y*	*z*
Nb	4.223718614085E−01	−4.536390078210E−01	−4.945076252371E−01
Nb	1.717784176120E−01	2.398917494018E−01	−4.948032456532E−01
Cl	−4.332217375744E−01	−2.299613183068E−01	4.479430447492E−01
Cl	3.183160082941E−01	4.553733052978E−03	4.608730558131E−01
O	−4.659030293543E−01	−8.472341704085E−02	3.713324243452E−01
O	2.941006447935E−01	−2.239725991816E−01	4.774391491541E−01
F	−4.373739326832E−01	−1.581887933056E−01	−3.654885043769E−01
F	2.856732781080E−01	2.068400114293E−01	3.713657990473E−01
F	−4.547020973042E−01	4.863404575349E−01	4.758256007658E−01
F	3.133654894660E−01	1.518878122977E−01	−3.706559558771E−01
F	3.841131158830E−01	−1.651736110091E−01	−4.417445088225E−01
F	4.080628529923E−01	4.048455108899E−01	−2.850491245341E−01
F	3.700960969055E−01	3.841623058914E−01	4.272545914293E−01
F	4.359702037101E−01	−3.145778522422E−01	3.075643169601E−01
F	4.698877655399E−01	−2.886594849586E−01	−3.950342350601E−01
F	2.206882882382E−01	8.594554346826E−02	−3.991169178898E−01
F	1.350169029018E−01	−4.864477525653E−02	−4.472338023059E−01
F	1.577639064852E−01	3.843277669442E−01	−2.860385799366E−01
F	1.847875032158E−01	1.166015602545E−01	3.024206866453E−01
F	2.020803353244E−01	−4.541564788684E−01	4.616003466913E−01
F	1.177301052609E−01	4.020119343878E−01	4.317451021735E−01
F	4.549391761342E−01	2.486022568836E−01	4.655983819191E−01

Lattice parameters and atomic coordinates of the optimized solid-state structure of $ClOF_2[RuF_6]$

Space group $Pna2_1$ (No. 33)
a = 15.05074141 Å, b = 5.17998973 Å, c = 8.40403396 Å, V = 655.201060 Å^3

Table S23. Atomic coordinates of the optimized structure of $ClOF_2[RuF_6]$.

Atom	*x*	*y*	*z*
Ru	−4.053834304356E−01	−3.953566547424E−01	2.829839056565E−01
Cl	−3.888657216199E−01	−1.409463153213E−01	−2.664503512197E−01
O	−3.397534963779E−01	−3.714661636097E−01	−2.495423382848E−01
F	−3.798890679127E−01	8.338666347106E−03	−9.724928461431E−02
F	−4.406445821760E−01	−2.419060475324E−01	4.799798859978E−01
F	−3.228162928861E−01	6.264745483152E−02	−3.557428378389E−01
F	4.931832811590E−01	−2.465517060903E−01	1.880289035556E−01
F	−3.737659248679E−01	4.573462064001E−01	9.078453534375E−02
F	−3.090496757371E−01	4.550994631337E−01	3.862675388484E−01
F	−4.769586951726E−01	3.124578320839E−01	3.352391347180E−01
F	−3.386109347436E−01	−1.053705567215E−01	2.370909078377E−01

Lattice parameters and atomic coordinates of the optimized solid-state structure of $ClOF_2[SbF_6]$

Space group *Pna*2_1 (No. 33)
a = 15.25805037 Å, *b* = 5.20681786 Å, *c* = 8.45561707 Å, *V* = 671.764017 Å^3

Table S24. Atomic coordinates of the optimized structure of $ClOF_2[SbF_6]$.

Atom	*x*	*y*	*z*
Sb	4.059946184780E−01	−1.085734416874E−01	4.534837768995E−01
Cl	−3.843194787899E−01	3.580474228628E−01	4.960763198278E−01
O	−3.358328181971E−01	1.286938819911E−01	4.815527957163E−01
F	−3.206003251700E−01	−4.369357943515E−01	−4.143463872163E−01
F	−4.893254416302E−01	−2.630290520820E−01	−4.609721116589E−01
F	4.382329359350E−01	−2.550039474741E−01	2.495975802462E−01
F	4.789600805580E−01	1.846893753411E−01	4.010829471841E−01
F	3.086262475702E−01	5.059884987259E−02	3.516879178981E−01
F	3.394975841132E−01	−4.086267402462E−01	4.980692531433E−01
F	3.782320725397E−01	3.394103852699E−02	−3.455846869947E−01
F	−3.757001365859E−01	−4.952806295422E−01	3.277225949545E−01

Lattice parameters and atomic coordinates of the optimized solid-state structures of $ClOF_2[TaF_6]$

Model 1 (full oxygen occupation on positions O(1A) and O(3A))

Energy difference relative to Model 5: 9.6 kJ/mol

Space group *Pna*2_1 (No. 33)
a = 30.40710140 Å, *b* = 5.22904067 Å, *c* = 8.46820243 Å, *V* = 1346.443932 Å^3

Table S25. Atomic coordinates of the optimized structure of $ClOF_2[TaF_6]$ (full oxygen occupation on positions O(1A) and O(3A)).

Atom	*x*	*y*	*z*
Ta	4.228748183414E−01	−4.745335309432E−01	−4.854440615671E−01
Ta	1.714705355059E−01	2.381716664734E−01	4.949702982445E−01
Cl	−4.315828845805E−01	−2.331892387869E−01	4.850429925984E−01
Cl	3.188610930987E−01	−8.931254857564E−03	4.547916139660E−01
O	−4.385298028561E−01	−1.446905476885E−01	−3.596913039092E−01
O	2.941042280161E−01	−2.365434783415E−01	4.661948515794E−01
F	−4.685402722415E−01	−9.279242654004E−02	3.763594765228E−01
F	−4.564732847694E−01	4.903087057983E−01	4.732358878672E−01
F	2.863473289977E−01	1.981380008449E−01	3.677497842375E−01
F	3.135579508882E−01	1.314030034252E−01	−3.750951810356E−01
F	3.830738300500E−01	−1.843826212448E−01	−4.447735784693E−01
F	4.069359722767E−01	3.892426377611E−01	−2.802886864492E−01
F	3.713120445901E−01	3.580095865255E−01	4.279384607143E−01
F	4.362076670358E−01	−3.430374919985E−01	3.118855888193E−01
F	4.228748183414E−01	−4.745335309432E−01	−4.854440615671E−01
F	1.714705355059E−01	2.381716664734E−01	4.949702982445E−01
F	−4.315828845805E−01	−2.331892387869E−01	4.850429925984E−01
F	3.188610930987E−01	−8.931254857564E−03	4.547916139660E−01
F	−4.385298028561E−01	−1.446905476885E−01	−3.596913039092E−01
F	2.941042280161E−01	−2.365434783415E−01	4.661948515794E−01
F	−4.685402722415E−01	−9.279242654004E−02	3.763594765228E−01
F	−4.564732847694E−01	4.903087057983E−01	4.732358878672E−01

Model 2 (full oxygen occupation on positions O(1A) and O(3B))

Energy difference relative to Model 5: 6.4 kJ/mol

Space group *Pna*2_1 (No. 33)
a = 30.45703786 Å, *b* = 5.26770955 Å, *c* = 8.40785604 Å, *V* = 1348.946579Å^3

Table S26. Atomic coordinates of the optimized structure of $ClOF_2[TaF_6]$ (full oxygen occupation on positions O(1A) and O(3B)).

Atom	*x*	*y*	*z*
Ta	4.222901365176E−01	−4.598849123116E−01	−4.837913362912E−01
Ta	1.707847088089E−01	2.334708493979E−01	4.952248110381E−01
Cl	−4.317713999744E−01	−2.315420539205E−01	4.859704092708E−01
Cl	3.176132809787E−01	5.427234922679E−02	4.419068745847E−01
O	−4.386878530455E−01	−1.427336787686E−01	−3.578644114272E−01
O	2.866433384170E−01	2.075208663360E−01	3.614898373137E−01
F	−4.685406623241E−01	−9.246144783253E−02	3.756137020128E−01
F	2.931867468320E−01	−2.187621499626E−01	4.681244440331E−01
F	−4.567435894686E−01	4.944879122683E−01	4.748234881952E−01
F	3.129992912914E−01	1.272015480960E−01	−3.711769139237E−01
F	3.836099674091E−01	−1.745400258153E−01	−4.488443529079E−01
F	4.067195250613E−01	4.031513027390E−01	−2.771893347121E−01
F	3.695991662039E−01	3.680472102534E−01	4.325573953582E−01
F	4.355612263479E−01	−3.469829735520E−01	3.079749814690E−01
F	4.700465007458E−01	−2.881429467087E−01	−3.903258924170E−01
F	2.203057025563E−01	7.885896978718E−02	−4.113046359878E−01
F	1.341545671618E−01	−5.638204664282E−02	−4.474139562861E−01
F	1.572101963533E−01	3.736762174989E−01	−2.933395509664E−01
F	1.831556507859E−01	1.042033920919E−01	2.924636702730E−01
F	2.013601725678E−01	−4.630788495274E−01	4.532164079796E−01
F	1.159847222577E−01	3.908234928526E−01	4.249305461210E−01
F	4.554812034081E−01	2.425668340235E−01	4.793738172703E−01

Model 3 (full oxygen occupation on positions O(1A) and O(3C))

Energy difference relative to Model 5: 7.9 kJ/mol

Space group $Pna2_1$ (No. 33)
a = 30.30441561 Å, b = 5.30144461 Å, c = 8.39435863 Å, V = 1348.613993 Å^3

Table S27. Atomic coordinates of the optimized structure of $ClOF_2[TaF_6]$ (full oxygen occupation on positions O(1A) and O(3C)).

Atom	*x*	*y*	*z*
Ta	4.226711960274E−01	−4.691457034604E−01	−4.925798243652E−01
Ta	1.712887891196E−01	2.437348699292E−01	−4.950583945174E−01
Cl	−4.307648110817E−01	−2.346042239848E−01	4.798865166984E−01
Cl	3.202992639418E−01	4.345680399707E−02	4.781406283122E−01
O	−4.382753738963E−01	−1.508280233500E−01	−3.630500499275E−01
O	3.134766708213E−01	1.328546522667E−01	−3.656288726913E−01
F	−4.675633650948E−01	−9.222984970652E−02	3.702798025859E−01
F	2.918995085979E−01	−2.173167674757E−01	4.648475279905E−01
F	−4.562417768672E−01	4.940211277671E−01	4.644800849989E−01
F	2.853359670321E−01	1.972634289371E−01	3.656772458098E−01
F	3.832701587509E−01	−1.848977495150E−01	−4.505793360676E−01
F	4.057558516247E−01	3.885029547760E−01	−2.889545706482E−01
F	3.696774503106E−01	3.667273038751E−01	4.203424306363E−01
F	4.364277153137E−01	−3.457420646494E−01	3.022710541251E−01
F	4.696414540369E−01	−2.975676369590E−01	−3.934892998738E−01
F	2.200020494038E−01	7.936855991747E−02	−4.022168689987E−01
F	1.334333852443E−01	−4.684402119718E−02	−4.510724930580E−01
F	1.552517142259E−01	3.788913315755E−01	−2.886033961672E−01
F	1.840160310968E−01	1.258468552408E−01	2.976413868436E−01
F	2.032720092745E−01	−4.580635846105E−01	4.661693272372E−01
F	1.169966599802E−01	4.019038571914E−01	4.240774270608E−01
F	4.562276377524E−01	2.377911433618E−01	4.698396740161E−01

Model 4 (full oxygen occupation on positions O(1B) and O(3A))

Energy difference relative to Model 5: 4.7 kJ/mol

Space group *Pna*2_1 (No. 33)
a = 30.63822175 Å, b = 5.21157319 Å, c = 8.47935533 Å, V = 1353.926944 Å^3

Table S28. Atomic coordinates of the optimized structure of $ClOF_2[TaF_6]$ (full oxygen occupation on positions O(1B) and O(3A)).

Atom	*x*	*y*	*z*
Ta	4.219653362156E−01	−4.551295623314E−01	−4.951760168421E−01
Ta	1.712651878335E−01	2.377434668366E−01	−4.956148391515E−01
Cl	−4.339979574936E−01	−2.300013377336E−01	4.490030298970E−01
Cl	3.176238975742E−01	2.418842090299E−03	4.617647324603E−01
O	−4.665214706466E−01	−8.491997238686E−02	3.718110592312E−01
O	2.935926898864E−01	−2.269530173419E−01	4.781741606746E−01
F	−4.383175732146E−01	−1.585487841952E−01	−3.650678439004E−01
F	−4.552068985717E−01	4.851674531254E−01	4.759628815176E−01
F	2.852096741278E−01	2.044889945360E−01	3.713472985348E−01
F	3.125549675282E−01	1.507102946588E−01	−3.706824524876E−01
F	3.838149433714E−01	−1.653714102240E−01	−4.416274677589E−01
F	4.086609038729E−01	4.044006921491E−01	−2.847090283306E−01
F	3.694673718497E−01	3.799416752193E−01	4.296824367510E−01
F	4.345478861820E−01	−3.169157479234E−01	3.055142636958E−01
F	4.699339894879E−01	−2.878267640441E−01	−3.991494866087E−01
F	2.206568025718E−01	8.078561336686E−02	−4.043996891577E−01
F	1.345934054870E−01	−5.281529639463E−02	−4.477487470538E−01
F	1.584032446165E−01	3.792944510710E−01	−2.853396146660E−01
F	1.832417904473E−01	1.167432682184E−01	2.999800357868E−01
F	2.016622277415E−01	−4.551669077969E−01	4.603804481228E−01
F	1.169942442160E−01	4.017054645262E−01	4.346796661705E−01
F	4.546797772095E−01	2.467612539089E−01	4.636351731147E−01

Model 5 (full oxygen occupation on positions O(1B) and O(3B))

Space group *Pna*2_1 (No. 33)
a = 30.69052327 Å, *b* = 5.25193804 Å, *c* = 8.42283279 Å, *V* = 1357.632001 Å^3

Table S29. Atomic coordinates of the optimized structure of $ClOF_2[TaF_6]$ (full oxygen occupation on positions O(1B) and O(3B)).

Atom	*x*	*y*	*z*
Ta	4.213576152608E−01	−4.416956311913E−01	−4.934392738326E−01
Ta	1.707350781252E−01	2.323614309034E−01	−4.953974850326E−01
Cl	−4.340045558084E−01	−2.292365487276E−01	4.494156272408E−01
Cl	3.165600162671E−01	6.204439997863E−02	4.474768258493E−01
O	−4.661938482657E−01	−8.613390642581E−02	3.694842704633E−01
O	2.855538753949E−01	2.107512106802E−01	3.651065317727E−01
F	−4.386505556627E−01	−1.550240981015E−01	−3.640727019383E−01
F	2.932831267261E−01	−2.125656221251E−01	4.783829339362E−01
F	−4.553379446638E−01	4.893762807856E−01	4.783666846370E−01
F	3.120724264864E−01	1.417383794873E−01	−3.668846665719E−01
F	3.842919433735E−01	−1.555695485061E−01	−4.442214627125E−01
F	4.087721508485E−01	4.197013528593E−01	−2.806089006868E−01
F	3.679311439194E−01	3.889358138032E−01	4.357551918744E−01
F	4.335875066438E−01	−3.194803555509E−01	3.019671642939E−01
F	4.703414007610E−01	−2.811425089803E−01	−4.022115491801E−01
F	2.207334501297E−01	7.661385025346E−02	−4.083198616927E−01
F	1.345638483284E−01	−5.721221188179E−02	−4.451012156257E−01
F	1.586738823221E−01	3.652129517579E−01	−2.806757215759E−01
F	1.821698455225E−01	1.135581926158E−01	2.986572166728E−01
F	2.010328589894E−01	−4.610228740436E−01	4.644463691373E−01
F	1.165743787632E−01	3.983138564731E−01	4.379173758496E−01
F	4.526744258573E−01	2.551881447279E−01	4.663766471219E−01

Model 6 (full oxygen occupation on positions O(1B) and O(3C))

Energy difference relative to Model 5: 8.7 kJ/mol

Space group *Pna*2_1 (No. 33)
a = 30.54138843 Å, *b* = 5.27351400 Å, *c* = 8.40013732 Å, *V* = 1352.929808 Å^3

Table S30. Atomic coordinates of the optimized structure of $ClOF_2[TaF_6]$ (full oxygen occupation on positions O(1B) and O(3C)).

Atom	*x*	*y*	*z*
Ta	4.212837457685E−01	−4.492359622129E−01	4.974228580069E−01
Ta	1.708930996086E−01	2.444614361563E−01	−4.860216625325E−01
Cl	−4.333224421775E−01	−2.320814559025E−01	4.440459609882E−01
Cl	3.187983166578E−01	5.477450663191E−02	4.845991571779E−01
O	−4.655068398381E−01	−8.544638660922E−02	3.658485392543E−01
O	3.122716234754E−01	1.483267494551E−01	−3.600019400217E−01
F	−4.384094052577E−01	−1.662917447577E−01	−3.679366289654E−01
F	2.913040498220E−01	−2.094432009773E−01	4.746438748036E−01
F	−4.554239861714E−01	4.870914184876E−01	4.670763152138E−01
F	2.839048682166E−01	2.037906915274E−01	3.707059802283E−01
F	3.838307661088E−01	−1.636065308125E−01	−4.460979634356E−01
F	4.074028282868E−01	4.047464705741E−01	−2.928442204225E−01
F	3.673454561180E−01	3.896770946774E−01	4.233909988486E−01
F	4.342849341029E−01	−3.170771235660E−01	2.957420988436E−01
F	4.696703506821E−01	−2.901712926897E−01	−4.053059782640E−01
F	2.202128001033E−01	8.027705282859E−02	−3.990258544139E−01
F	1.337401473368E−01	−4.623654361654E−02	−4.493273205822E−01
F	1.564326425644E−01	3.738810772964E−01	−2.763722690481E−01
F	1.829958689673E−01	1.376034984466E−01	3.033650451002E−01
F	2.024690733279E−01	−4.532141730656E−01	4.760998212002E−01
F	1.170537953834E−01	4.110158049705E−01	4.369969951256E−01
F	4.527992459758E−01	2.510748996249E−01	4.554161928946E−01

Lattice parameters and atomic coordinates of the optimized solid-state structure of $ClOF_2[OsF_6]$

Space group $Pna2_1$ (No. 33)
a = 15.25805037 Å, b = 5.20681786 Å, c = 8.45561707 Å, V = 671.764017 Å^3

Table S31. Atomic coordinates of the optimized structure of $ClOF_2[OsF_6]$.

Atom	*x*	*y*	*z*
Os	4.082386746445E−01	3.910123810543E−01	−4.977230922155E−01
Cl	1.097800733173E−01	−4.066583686038E−01	4.727802130408E−01
O	1.228511277670E−01	−4.952701100265E−01	−3.693247640527E−01
F	1.802746547484E−01	4.418563801279E−01	3.595521715724E−01
F	1.662636029880E−01	−1.457100526718E−01	4.594132356443E−01
F	3.101124312580E−01	−4.532033498061E−01	−3.991126071943E−01
F	3.407865298306E−01	9.977079523670E−02	4.645133892418E−01
F	4.802766000225E−01	−3.154966924896E−01	−4.542567016032E−01
F	3.756433868229E−01	−4.827545026842E−01	2.990204417516E−01
F	−4.875221667650E−01	2.382143857115E−01	4.054495199134E−01
F	4.447808671667E−01	2.539359565969E−01	−2.947018060987E−01

Lattice parameters and atomic coordinates of the optimized solid-state structure of $ClOF_2[IrF_6]$

Space group $Pna2_1$ (No. 33)
a = 15.09574775 Å, b = 5.13646909 Å, c = 8.31062347 Å, V = 644.396118 Å^3

Table S32. Atomic coordinates of the optimized structure of $ClOF_2[IrF_6]$.

Atom	*x*	*y*	*z*
Ir	4.048234990928E−01	3.814781207281E−01	−4.774094027396E−01
Cl	1.124653105106E−01	−3.637790224478E−01	−4.268981346726E−01
O	1.617912507093E−01	−1.319961740300E−01	−4.442404875151E−01
F	1.759311079238E−01	4.319746985017E−01	−3.315817462498E−01
F	1.234339077147E−01	4.816047346509E−01	4.033656108270E−01
F	3.684122913874E−01	−4.753185385918E−01	−2.807642728658E−01
F	3.053295049777E−01	−4.735738294498E−01	4.183902933915E−01
F	3.432311042832E−01	8.194495219675E−02	−4.346614076906E−01
F	4.444246755472E−01	2.312461746924E−01	3.214124682089E−01
F	−4.921152000784E−01	2.432560317700E−01	−3.737800439817E−01
F	4.707369954925E−01	−3.200142522967E−01	4.729771232878E−01

Comparison of low-resolution Raman spectra of $ClOF_2[MF_6]$ compounds (M = V, Nb, Ta, Ru, Os, Ir, P, Sb)

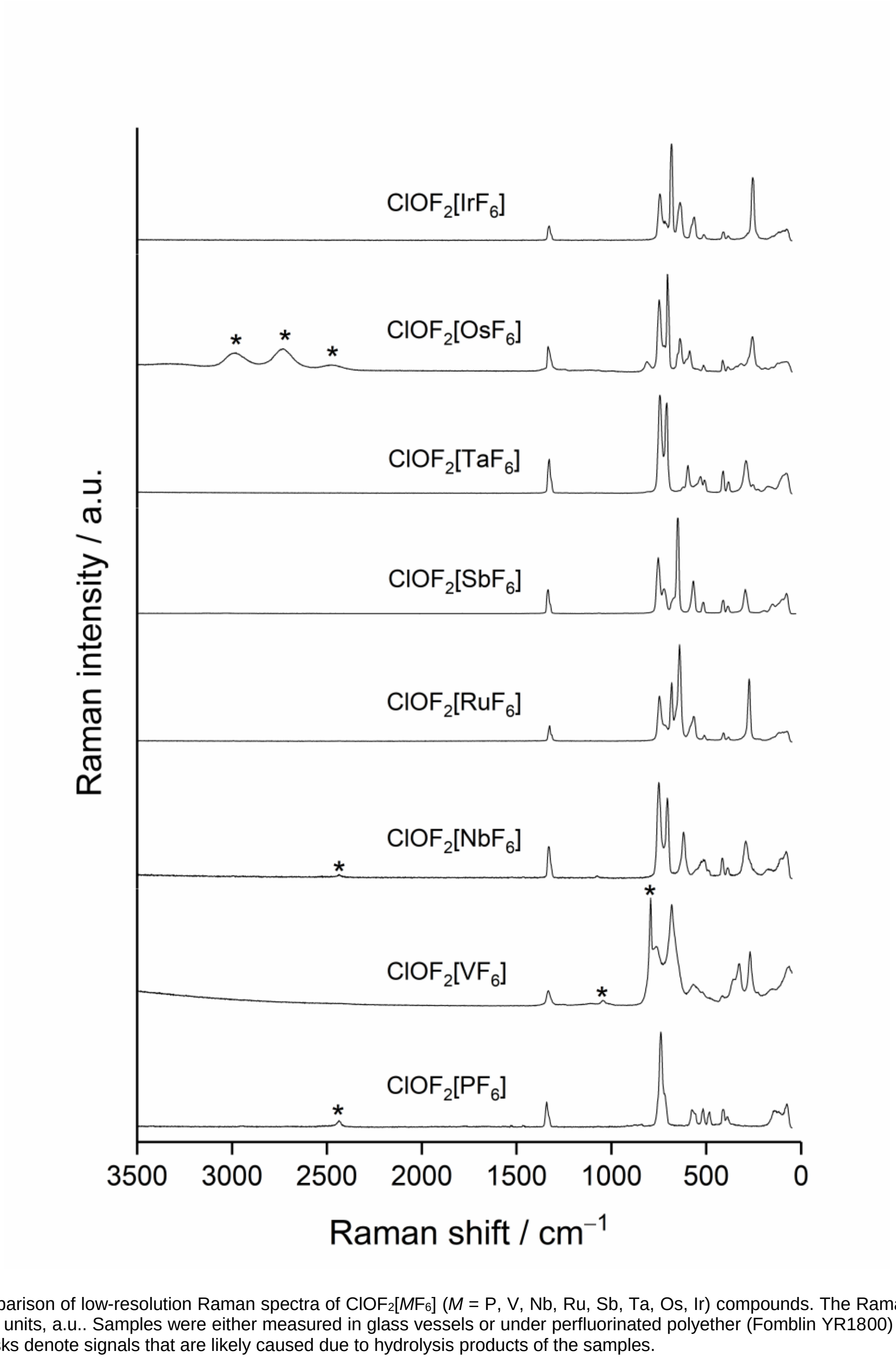

Figure S5. Comparison of low-resolution Raman spectra of $ClOF_2[MF_6]$ (M = P, V, Nb, Ru, Sb, Ta, Os, Ir) compounds. The Raman intensities are given in arbitrary units, a.u.. Samples were either measured in glass vessels or under perfluorinated polyether (Fomblin YR1800) on a microscope slide. The asterisks denote signals that are likely caused due to hydrolysis products of the samples.

Raman spectra and band assignments for the calculated Raman spectra of $ClOF_2[MF_6]$ (*M* = V, Nb, Ta, Ru, Os, Ir, P, Sb)

Raman spectra of $ClOF_2[PF_6]$

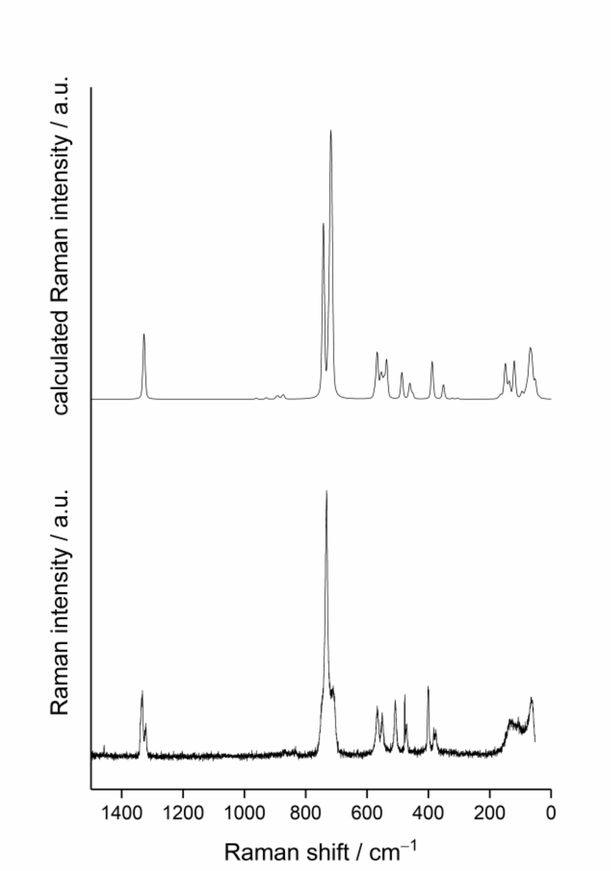

Figure S6. Comparison of the experimental high-resolution and calculated solid-state (DFT-PBE0/TZVP) Raman spectra of $ClOF_2[PF_6]$. The Raman intensities are given in arbitrary units, a.u..

Table S33. Band assignments for the calculated Raman spectrum (>300 cm^{-1}) of $ClOF_2[PF_6]$. The notation for band assignments is the following: *ν* – stretching, *δ* – deformation, s – symmetric, as – asymmetric.

ν (calculated) / cm^{-1}	*ν* (experimental) / cm^{-1}	Assignment
1329-1327	1332, 1321	*ν*(ClO) – Stretching of the O atom of the $ClOF_2^+$ cation
961-872	not observed	*ν*(ClF_2) + *ν*(PF_6) –Stretching of F atoms of the $ClOF_2^+$ cation coupled with stretching modes of the $[PF_6]^-$ anion
748-742	732	$\nu_s(ClF_2)$ + *ν*(PF_6) – Symmetric stretching of F atoms of the $ClOF_2^+$ cation coupled with stretching of the $[PF_6]^-$ anion
727-715	710	$\nu_{as}(ClF_2)$ + *ν*(PF_6) – Asymmetric stretching of F atoms of the $ClOF_2^+$ cation coupled with stretching of the $[PF_6]^-$ anion
576-563	566	$\nu_{as}(PF_6)$ – Asymmetric stretching of the $[PF_6]^-$ anion
558-536	551	*δ*(PF_6) – Scissoring of the $[PF_6]^-$ anion
495-486	508	*δ*($ClOF_2$) + *δ*(PF_6) – Umbrella bending of the $ClOF_2^+$ cation coupled with scissoring of the $[PF_6]^-$ anion
464-451	478, 471	*δ*(PF_6) – Wagging of the $[PF_6]^-$ anion
388-386	401	*δ*(ClO) – Scissoring of the O atom of the $ClOF_2^+$ cation
352-350	382	*δ*(ClF_2) – Scissoring of the F atoms of the $ClOF_2^+$ cation
322-303	375	*δ*(PF_6) – Twisting of the $[PF_6]^-$ anion

Raman spectra of $ClOF_2[VF_6]$

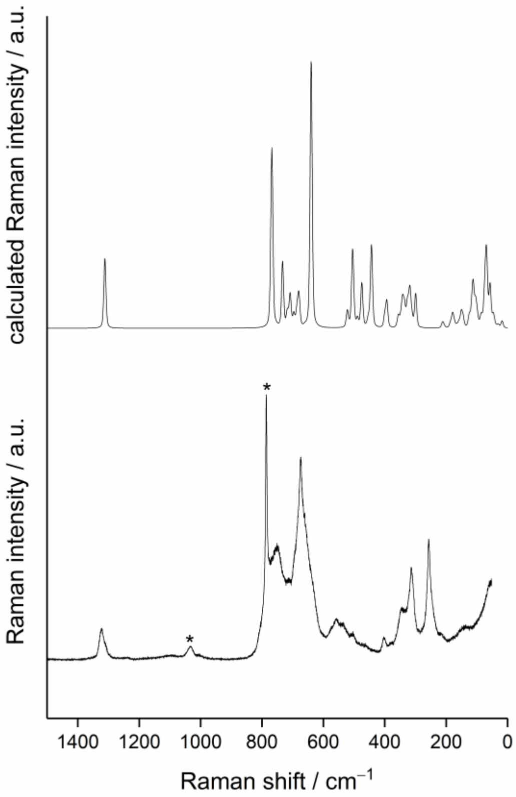

Figure S7. Comparison of the experimental high-resolution and calculated solid-state (DFT-PBE0/TZVP) Raman spectra of $ClOF_2[VF_6]$. The bands marked with an asterisk are likely caused by hydrolysis products. The Raman intensities are given in arbitrary units, a.u..

Table S34. Band assignments for the calculated Raman spectrum (>300 cm^{-1}) of $ClOF_2[VF_6]$. The notation for band assignments is the following: *v* – stretching, *δ* – deformation, s – symmetric, as – asymmetric.

v (calculated) / cm^{-1}	*v* (experimental) / cm^{-1}	Assignment
1312-1310	1322	*v*(ClO) – Stretching of the O atom of the $ClOF_2^+$ cation
798-642	750, 673	*v*(ClF_2) + *v*(VF_6) –Stretching of F atoms of the $ClOF_2^+$ cation coupled with stretching modes of the $[VF_6]^-$ anion
521-434	558, 506	*δ*($ClOF_2$) + *δ*(VF_6) – Umbrella bending of the $ClOF_2^+$ cation coupled with scissoring of the $[VF_6]^-$ anion
401-399	401	*δ*(ClO) + *δ*(VF_6) – Scissoring of the O atom of the $ClOF_2^+$ cation coupled with scissoring of the $[VF_6]^-$ anion
361-343	345	*δ*(ClF_2) + *δ*(VF_6) – Scissoring of the F atom of the $ClOF_2^+$ cation coupled with scissoring of the $[VF_6]^-$ anion
331-302	313, 256	*δ*($ClOF_2$) + *δ*(VF_6) – Scissoring of the O and F atoms of the $ClOF_2^+$ cation coupled with scissoring of the $[VF_6]^-$ anion

Raman spectrum of $ClOF_2[NbF_6]$

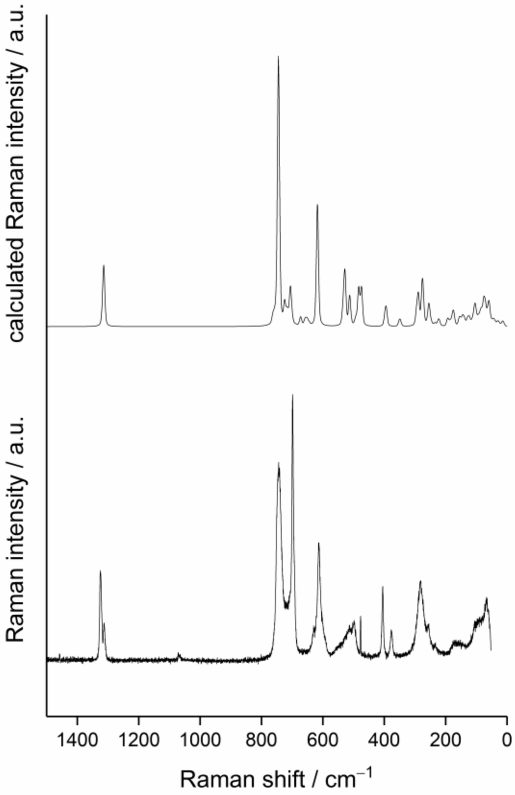

Figure S8. Comparison of the experimental high-resolution and calculated solid-state (DFT-PBE0/TZVP) Raman spectra of $ClOF_2[NbF_6]$. The Raman intensities are given in arbitrary units, a.u..

Table S35. Band assignments for the calculated Raman spectrum (>300 cm^{-1}) of $ClOF_2[NbF_6]$. The notation for band assignments is the following: *ν* – stretching, *δ* – deformation, s – symmetric, as – asymmetric.

ν (calculated) / cm^{-1}	*ν* (experimental) / cm^{-1}	Assignment
1317-1313	1325, 1313	*ν*(ClO) – Stretching of the O atom of the $ClOF_2^+$ cation
747-745	744	$\nu_s(ClF_2)$ – Symmetric stretching of F atoms of the $ClOF_2^+$ cation
738-705	not resolved due to overlapping bands	ν_{as}(ClF2) – Asymmetric stretching of F atoms of the $ClOF_2^+$ cation
701-510	698, 613	$\nu(NbF_6)$ – Stretching of the $[NbF_6]^-$ anion
493-490	499	$\delta(ClOF_2)$ – Umbrella bending of the $ClOF_2^+$ cation
483-474	477	$\delta(ClOF_2) + \nu(NbF_6)$ – Umbrella bending of the $ClOF_2^+$ cation coupled with stretching of the $[NbF_6]^-$ anion
398-393	405	*δ*(ClO) – Scissoring of the O atom of the $ClOF_2^+$ cation
351-347	377	$\delta(ClF_2)$ – Scissoring of the F atoms of the $ClOF_2^+$ cation

Raman spectra of $ClOF_2[RuF_6]$

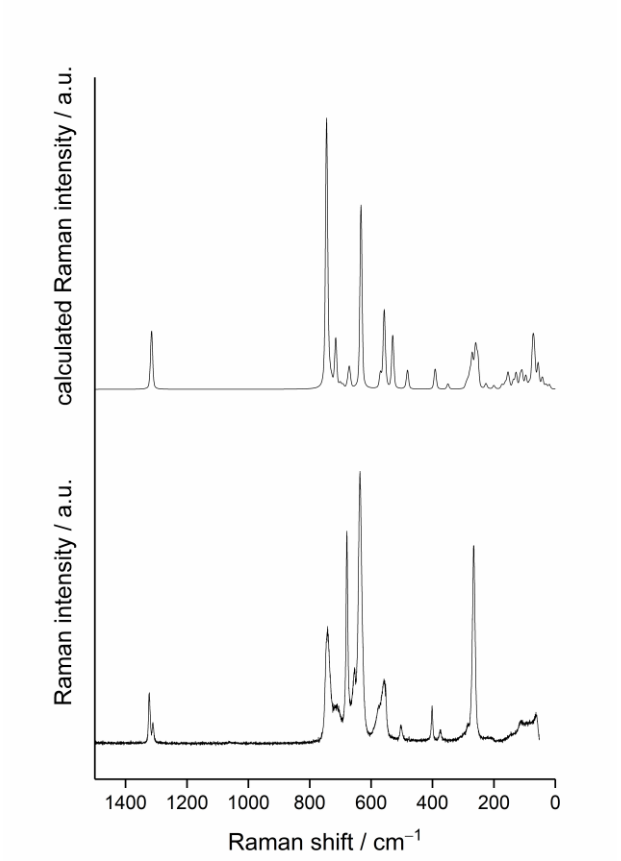

Figure S9. Comparison of the experimental high-resolution and calculated solid-state (DFT-PBE0/TZVP) Raman spectra of $ClOF_2[RuF_6]$. The Raman intensities are given in arbitrary units, a.u..

Table S36. Band assignments for the calculated Raman spectrum (>300 cm^{-1}) of $ClOF_2[RuF_6]$. The notation for band assignments is the following: *v* – stretching, *δ* – deformation, s – symmetric, as – asymmetric.

v (calculated) / cm^{-1}	*v* (experimental) / cm^{-1}	Assignment
1317-1315	1323, 1311	*v*(ClO) – Stretching of the O atom of the $ClOF_2^+$ cation
756-529	742, 714, 679, 654, 637, 558	*v*(ClF_2) + *v*(RuF_6) –Stretching of F atoms of the $ClOF_2^+$ cation coupled with stretching modes of the $[RuF_6]^-$ anion
491-481	503	*δ*($ClOF_2$) + *δ*(RuF_6) – Umbrella bending of the $ClOF_2^+$ cation coupled with scissoring of the $[RuF_6]^-$ anion
392-390	401	*δ*(ClO) – Scissoring of the O atom of the $ClOF_2^+$ cation
350-349	375	*δ*(ClF_2) – Scissoring of the F atoms of the $ClOF_2^+$ cation

Raman spectra of $ClOF_2[SbF_6]$

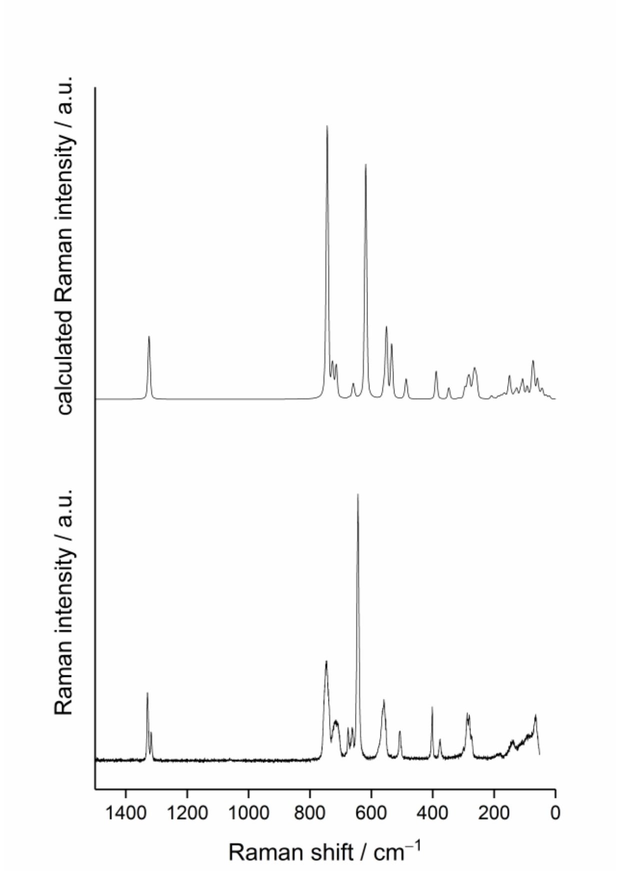

Figure S10. Comparison of the experimental high-resolution and calculated solid-state (DFT-PBE0/TZVP) Raman spectra of $ClOF_2[SbF_6]$. The Raman intensities are given in arbitrary units, a.u..

Table S37. Band assignments for the calculated Raman spectrum (>300 cm^{-1}) of $ClOF_2[SbF_6]$. The notation for band assignments is the following: *ν* – stretching, *δ* – deformation, s – symmetric, as – asymmetric.

ν (calculated) / cm^{-1}	ν (experimental) / cm^{-1}	Assignment
1325-1324	1330, 1318	$\nu(ClO)$ – Stretching of the O atom of the $ClOF_2^+$ cation
751-743	747	$\nu_s(ClF_2)$ – Symmetric stretching of F atoms of the $ClOF_2^+$ cation
727-714	715	$\nu_{as}(ClF_2)$ – Asymmetric stretching of F atoms of the $ClOF_2^+$ cation
688-533	675, 662, 643, 560	$\nu(SbF_6)$ –Stretching of the $[SbF_6]^-$ anion
494-484	507	$\delta(ClOF_2)$ – Umbrella bending of the $ClOF_2^+$ cation
389-388	402	$\delta(ClO)$ – Scissoring of the O atom of the $ClOF_2^+$ cation
348-347	377	$\delta(ClF_2)$ – Scissoring of the F atoms of the $ClOF_2^+$ cation
316-303	288, 281	$\delta(SbF_6)$ – Scissoring of the $[SbF_6]^-$ anion

Raman spectrum of $ClOF_2[TaF_6]$

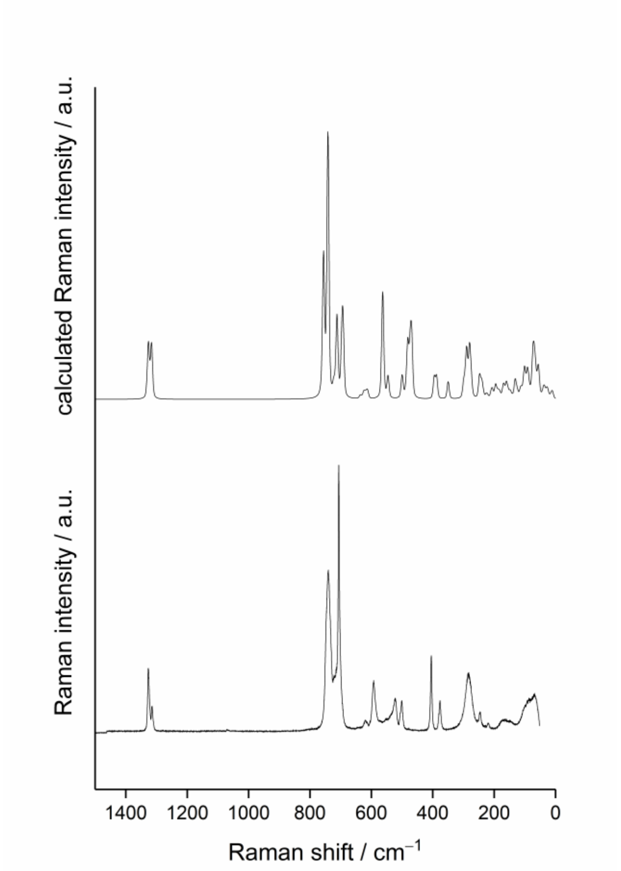

Figure S11. Comparison of the experimental high-resolution and calculated solid-state (DFT-PBE0/TZVP) Raman spectra of $ClOF_2[TaF_6]$. The Raman intensities are given in arbitrary units, a.u..

Table S38. Band assignments for the calculated Raman spectrum (>300 cm^{-1}) of $ClOF_2[TaF_6]$. The notation for band assignments is the following: v – stretching, δ – deformation, s – symmetric, as – asymmetric.

v (calculated) / cm^{-1}	v (experimental) / cm^{-1}	Assignment
1330-1316	1327, 1315	v(ClO) – Stretching of the O atom of the $ClOF_2^+$ cation
765-741	741	$v_s(ClF_2)$ – Symmetric stretching of F atoms of the $ClOF_2^+$ cation
722-711	not resolved due to overlapping bands	$v_{as}(ClF_2)$ – Asymmetric stretching of F atoms of the $ClOF_2^+$ cation
699-546	706, 617, 592, 522	$v(TaF_6)$ – Stretching of the $[TaF_6]^-$ anion
500-468	500	$\delta(ClOF_2)$ + $v(TaF_6)$ – Umbrella bending of the $ClOF_2^+$ cation coupled with stretching of the $[TaF_6]^-$ anion
395-388	404	δ(ClO) – Scissoring of the O atom of the $ClOF_2^+$ cation
350-349	376	$\delta(ClF_2)$ – Scissoring of the F atoms of the $ClOF_2^+$ cation

Raman spectra of $ClOF_2[OsF_6]$

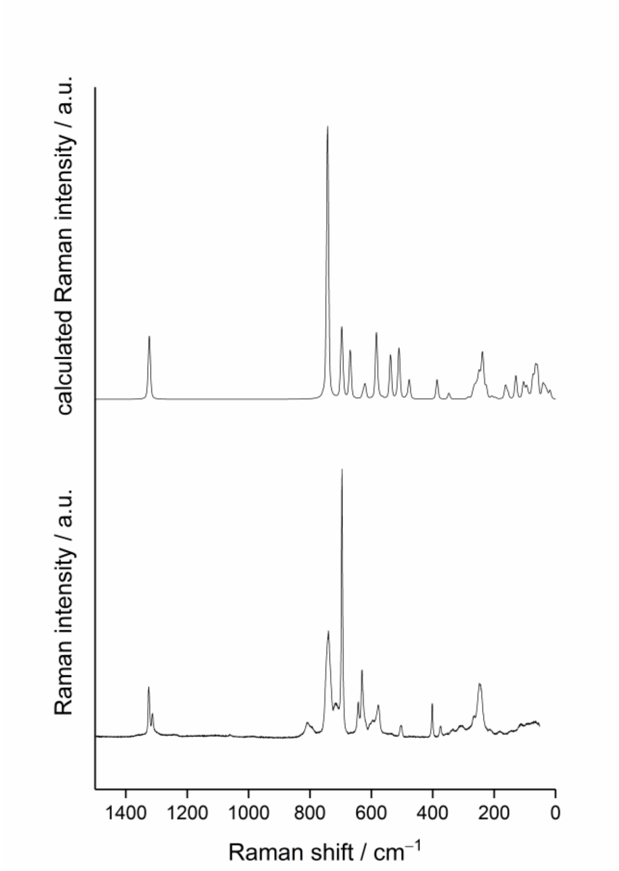

Figure S12. Comparison of the experimental high-resolution and calculated solid-state (DFT-PBE0/TZVP) Raman spectra of $ClOF_2[OsF_6]$. The Raman intensities are given in arbitrary units, a.u..

Table S39. Band assignments for the calculated Raman spectrum (>300 cm^{-1}) of $ClOF_2[OsF_6]$. The notation for band assignments is the following: v – stretching, δ – deformation, s – symmetric, as – asymmetric.

v (calculated) / cm^{-1}	v (experimental) / cm^{-1}	Assignment
1329-1323	1325, 1313	v(ClO) – Stretching of the O atom of the $ClOF_2^+$ cation
757-735	740	$v_s(ClF_2)$ – Symmetric stretching of F atoms of the $ClOF_2^+$ cation
699-692	716	$v_{as}(ClF_2)$ – Asymmetric stretching of F atoms of the $ClOF_2^+$ cation
673-510	696, 643, 631, 577	$v(OsF_6)$ – Stretching of the $[OsF_6]^-$ anion
486-476	503	$\delta(ClOF_2)$ – Umbrella bending of the $ClOF_2^+$ cation
386	402	δ(ClO) – Scissoring of the O atom of the $ClOF_2^+$ cation
348-347	375	$\delta(ClF_2)$ – Scissoring of the F atoms of the $ClOF_2^+$ cation

Raman spectrum of $ClOF_2[IrF_6]$

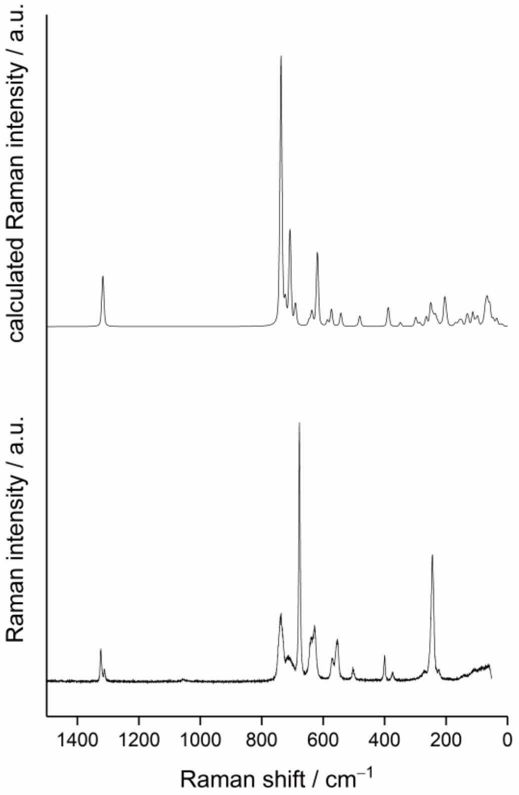

Figure S13. Comparison of the experimental high-resolution and calculated solid-state (DFT-PBE0/TZVP) Raman spectra of $ClOF_2[IrF_6]$. The Raman intensities are given in arbitrary units, a.u..

Table S40. Band assignments for the calculated Raman spectrum (>300 cm^{-1}) of $ClOF_2[IrF_6]$. The notation for band assignments is the following: *v* – stretching, *δ* – deformation, s – symmetric, as – asymmetric.

v (calculated) / cm^{-1}	*v* (experimental) / cm^{-1}	Assignment
1319-1317	1324, 1313	*v*(ClO) – Stretching of the O atom of the $ClOF_2^+$ cation
743-737	737	$v_s(ClF_2)$ – Symmetric stretching of F atoms of the $ClOF_2^+$ cation
727-708	713	$v_{as}(ClF_2)$ – Asymmetric stretching of F atoms of the $ClOF_2^+$ cation
692-542	677, 639, 628, 570, 555	$v(IrF_6)$ – Stretching of the $[IrF_6]^-$ anion
491-481	503	$\delta(ClOF_2)$ – Umbrella bending of the $ClOF_2^+$ cation
390-384	400	*δ*(ClO) – Scissoring of the O atom of the $ClOF_2^+$ cation
349	374	$\delta(ClF_2)$ – Scissoring of the F atoms of the $ClOF_2^+$ cation

References

[1] R. D. Shannon, *Acta Crystallogr., Sect. A: Cryst. Phys., Diffr., Theor. Gen. Crystallogr.* **1976**, *32*, 751–767.
[2] D. S. Brock, J. J. Casalis de Pury, H. P. A. Mercier, G. J. Schrobilgen, B. Silvi, *Inorg. Chem.* **2010**, *49*, 6673–6689.
[3] A. J. Karttunen, T. Tynell, M. Karppinen, *J. Phys. Chem. C* **2015**, *119*, 13105–13114.
[4] V. Sivchik, R. K. Sarker, Z.-Y. Liu, K.-Y. Chung, E. V. Grachova, A. J. Karttunen, P.-T. Chou, I. O. Koshevoy, *Chem. Eur. J.* **2018**, *24*, 11475–11484.
[5] M. S. Kuklin, A. J. Karttunen, *J. Phys. Chem. C* **2018**, *122*, 24949–24957.
[6] F. Weigend, R. Ahlrichs, *Phys. Chem. Chem. Phys.* **2005**, *7*, 3297–3305.
